# Structure and design of Langya virus glycoprotein antigens

**DOI:** 10.1073/pnas.2314990121

**Published:** 2024-04-09

**Authors:** Zhaoqian Wang, Matthew McCallum, Lianying Yan, Cecily A. Gibson, William Sharkey, Young-Jun Park, Ha V. Dang, Moushimi Amaya, Ashley Person, Christopher C. Broder, David Veesler

**Affiliations:** ^a^Department of Biochemistry, University of Washington, Seattle, WA 98195; ^b^Department of Microbiology and Immunology, Uniformed Services University, Bethesda, MD 20814; ^c^HHMI, Seattle, WA 98195

**Keywords:** Langya virus, Henipavirus, CryoEM, vaccine design, antibodies

## Abstract

Langya virus (LayV) is a recently discovered henipavirus (HNV), isolated from febrile patients in China. HNV entry into host cells is mediated by the attachment (G) and fusion (F) glycoproteins which are the main targets of neutralizing antibodies. We show here that the LayV F and G glycoproteins promote membrane fusion with human, mouse, and hamster target cells using a different, yet unknown, receptor than Nipah virus (NiV) and Hendra virus (HeV) and that NiV- and HeV-elicited monoclonal and polyclonal antibodies do not cross-react with LayV F and G. We determined cryoelectron microscopy structures of LayV F, in the prefusion and postfusion states, and of LayV G, revealing their conformational landscape and distinct antigenicity relative to NiV and HeV. We computationally designed stabilized LayV G constructs and demonstrate the generalizability of an HNV F prefusion-stabilization strategy. Our data will support the development of vaccines and therapeutics against LayV and closely related HNVs.

## Significance

Langya virus (LayV) was discovered in febrile patients in China for which no countermeasures exist. We describe the architecture and antigenicity of the LayV fusion (F) and attachment (G) glycoproteins, suggesting that vaccines and therapeutics currently being developed against Nipah virus (NiV)/Hendra virus (HeV) will be ineffective for LayV. We designed stabilized version of each glycoprotein to support the development of vaccines and therapeutics against these pathogens.

Nipah virus (NiV) and Hendra virus (HeV) are bat-borne zoonotic pathogens of the Henipavirus (HNV) genus causing severe encephalitis and respiratory symptoms in humans (1). NiV has spilled over to humans on a regular basis, and the detection of HNVs in several continents underscores their global distribution (2–9). To date, no approved vaccines or therapeutics exist for use in humans against any HNVs (10).

HNV infections are facilitated by the attachment glycoprotein (G) and the fusion glycoprotein (F). Upon G-mediated receptor engagement, F is triggered and promotes membrane fusion and viral entry (11–15). NiV, HeV, Ghanaian virus (GhV), and Cedar virus (CedV) utilize a subset of ephrin receptors to gain entry into host cells (16–20). F and G are the targets of neutralizing antibodies which are correlates of protection against NiV and HeV (21–28).

Although most HNVs are bat-borne, Mojiang virus (MojV) was discovered in rats (29), whereas Gamak (GAKV) and Daeryong (DARV) HNVs were identified in shrews (30). Langya virus (LayV) was recently found in shrews and isolated from febrile patients in China (31), representing the first human-infecting HNV from shrew origin. LayV, MojV, GAKV, and DARV phylogenetically cluster together and are distantly related to bat-borne HNVs (30–32). The host receptor(s) used by MojV, LayV, GAKV, and DARV remain elusive (32).

LayV F and G are closely related to MojV F and G but distantly related to their NiV and HeV counterparts (*SI Appendix*, Fig. S1). The scarcity of structural and functional information for LayV and MojV F and G combined with their divergence from NiV/HeV F and G limits our ability to assess conserved and divergent architectural traits for developing countermeasures and antigen design strategy broadly applicable to HNVs (32–34). Here, we determined cryoEM (cryoelectron microscopy) structures of LayV F in the prefusion and postfusion conformations, revealing the refolding events underlying membrane fusion. We show the broad applicability of an HNV F prefusion-stabilization strategy which will pave the way for vaccine development efforts. NiV/HeV-elicited polyclonal and monoclonal antibodies (mAbs) did not cross-react with LayV F and G, suggesting that vaccines and therapeutics currently being developed against NiV/HeV will be ineffective for LayV. We identified a MojV F-directed mAb (4G5) binding to LayV F, and unveiled the molecular basis of recognition, as well as a MojV G-directed mAb (2B2) cross-reacting with LayV G. We computationally designed stabilized LayV G constructs and determined cryoEM structures of LayV G, which adopts a unique conformation, defining another snapshot of the host invasion process. Our data provide blueprints of the two key targets of LayV vaccine design for developing countermeasures against this emerging pathogen.

## Functional Assessment of the LayV F and G Glycoproteins

**Fig. 1.**
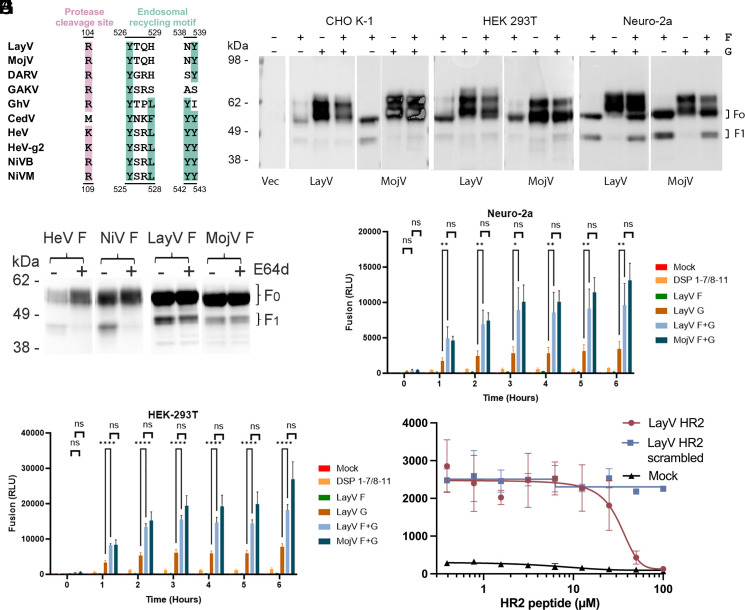
Functional assessment of the LayV F and G glycoproteins. (*A*) HNV F sequence alignment focused on the activating protease cleavage site and the endosomal recycling motif present in NiV/HeV F, with LayV (UUV47205.1), MojV (YP_009094094.1), DARV (QYO90531.1), GAKV (QYO90517.1), GhV (YP_009091837.1), CedV (YP_009094085.1), HeV (AEQ38140.1), HeV-g2 (QYC64604.1), NiVM (AAK50553.1), and NiVB (AEZ01396.1). NiVM: NiV-Malaysia; NiVB: NiV-Bangladesh. (*B*–*D*) Assessment of HNV F proteolytic cleavage in CHO-K1 (*B*), HEK293T (*C*) or mouse Neuro-2a cells (*D*) using Western blot with rabbit anti-S peptide:HRP antibodies. Transfected plasmids are indicated above each lane. (*E*) Assessment of HNV F proteolytic cleavage in mouse Neuro-2a cells in the presence or absence of 50 µM of the cysteine protease inhibitor E64d using Western blot with rabbit anti-S peptide:HRP antibodies. (*F* and *G*) HNV F/G-mediated membrane fusion. Effector Neuro-2a cells were transfected with LayV F, LayV G, LayV F and LayV G, MojV F, MojV G or MojV F and MojV G along with the DSP 1 to 7 plasmid. Target (HEK293T or Neuro-2a) cells were transfected with DSP 8-11 for luciferase detection upon cell/cell fusion. DSP 1 to 7/8 to 11: cells transfected with the split luciferase plasmids only. Mock: cells transfected with an empty pcDNA3.1 plasmid. ****P < 0.001; **P < 0.01; *P < 0.05; ns: not significant; multiple unpaired *t* test, n = 4 biological replicates. (*H*) Concentration-dependent inhibition of cell–cell fusion by a LayV F HR2 peptide or a scrambled peptide of identical composition using HEK293T target cells (n = 3 biological replicates). Effector Neuro-2a cells were cotransfected with LayV F, LayV G, and a DSP 1 to 7 plasmid. Target HEK293T cells were transfected with DSP 8 to 11. Mock: cells transfected with an empty pcDNA3.1 plasmid. Error bars: SD in *F*–*H*.

LayV F possesses a putative cleavage site at residue 104 (R104), as is the case for NiV (R109) and HeV (K109), but lacks the canonical YXXФ motif and one out of the two downstream tyrosine residues present in the NiV/HeV F C-terminal cytoplasmic domains, which promote endosomal recycling and in turn cathepsin L-mediated cleavage (11, 14, 35) (Fig. 1*A*). Nevertheless, we observed that transient transfection of LayV F or LayV F and G in CHO-K1, HEK293T or mouse Neuro-2a cells resulted in production of the F_0_ precursor and proteolytically cleaved F_1_ (and F_2_) with approximately similar ratio to that observed for other HNVs (Fig. 1 *B*–*D*). These data are reminiscent of findings made with MojV F (36) and suggest that the LayV/MojV F proteolytic cleavage pathway is likely distinct from that described for NiV and HeV F. To test this hypothesis, we analyzed the effect of the general cysteine protease inhibitor Aloxistatin (E64d) on proteolytic processing of LayV F, MojV F, NiV F, and HeV F upon transient transfection of mouse Neuro-2a cells. Although E64d had no effect on the ratio of cleaved/uncleaved F for LayV and MojV, cleavage of NiV F and HeV F was abrogated, strongly suggesting that a distinct protease is involved in F proteolytic processing for this highly divergent HNV subgroup (Fig. 1*E*).

We subsequently assayed the ability of LayV F and G to promote cell–cell fusion using a split luciferase system. Transient transfection of Neuro-2a effector cells with LayV F and G resulted in detectable fusion with target HEK293T or Neuro-2a cells (Fig. 1 *F* and *G*). Furthermore, a LayV F HR2 (heptad repeat 2) peptide, but not a scrambled peptide with identical composition, inhibited LayV F/G-mediated fusion in a concentration-dependent manner, demonstrating that the observed membrane fusion was promoted by F specifically (Fig. 1*H*). These data show that the LayV F and G glycoproteins are fusion-competent although they promote cell–cell fusion much more weakly than their NiV/HeV counterparts, as was the case for MojV F and G (32, 36).

## Structures of LayV F in the Prefusion and Postfusion Conformations

To understand the architecture of LayV F, we produced a LayV F glycoprotein ectodomain construct C-terminally fused to a GCN4 trimerization motif (13, 21, 22). Using EM imaging of negatively stained samples, we observed that LayV F formed well-folded compact homotrimers, typical of the prefusion state, but that the protein spontaneously refolded to the postfusion state (*SI Appendix*, Fig. S2). As a result, we collected two cryoEM datasets 4 mo apart and determined structures of prefusion F and postfusion F at 2.5 Å and 3.9 Å resolution, respectively (*SI Appendix*, Fig. S3 and Table 1).

**Fig. 2.**
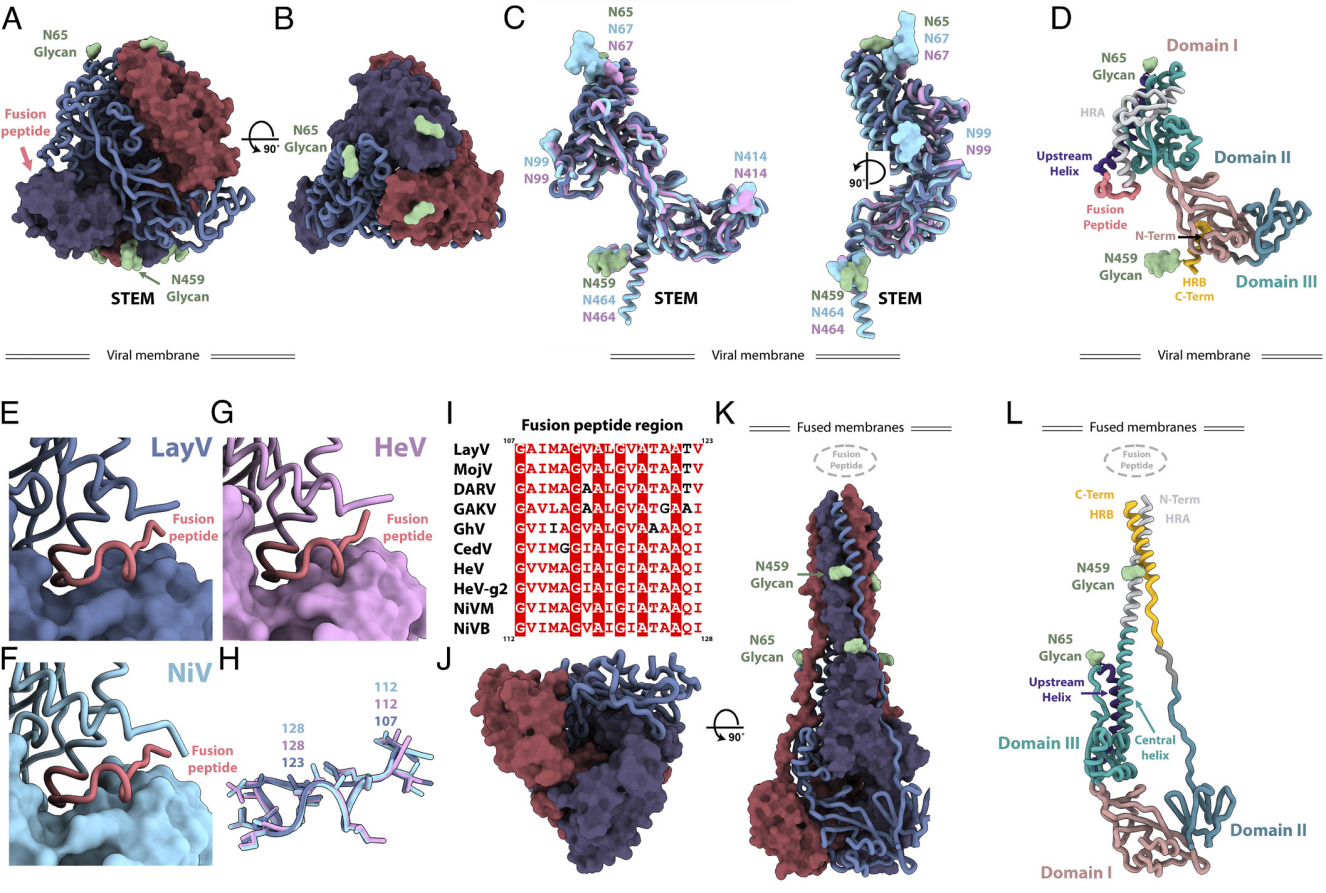
Structures of LayV F in the prefusion and postfusion conformations. (*A* and *B*) Prefusion LayV F trimer structure in two orthogonal orientations with each protomer colored distinctly. (*C*) Superimposition of LayV F (dark blue) with NiV F (cyan, PDB 7KI4) and HeV F (pink, PDB 7KI6). (*D*) Ribbon diagram of a LayV F protomer in the prefusion state colored and annotated by functional domains as defined in ref. 22. (*E*–*G*) Zoomed-in views of LayV F (*E*, blue), NiV F (*F*, cyan), and HeV F (*G*, pink) trimers focused on the fusion peptide (salmon ribbons). A neighboring protomer is rendered as a surface. (*H*) Superimposition of LayV F (blue), NiV F (cyan), and HeV F (pink) fusion peptides with side chains shown as sticks. (*I*) Sequence alignment of HNV F fusion peptides. NiVM: NiV-Malaysia; NiVB: NiV-Bangladesh. (*J* and *K*) Postfusion LayV F structure in two orthogonal orientations. (*L*) Ribbon diagram of a LayV F protomer in the postfusion state colored and annotated by functional domains.

Prefusion LayV F folds as a ~90 Å-high and ~90 Å-wide pyramidal-shaped trimer, typical of prefusion HNV F (Fig. 2 *A* and *B*). Although LayV F shares only 44% amino acid sequence identity with NiV F, they adopt strikingly similar tertiary and quaternary structures (12, 13, 21, 22) (Fig. 2 *A*–*D*). A LayV F protomer can be superimposed to NiV F with a rmsd of 2.3 Å over 432 Cα pairs as compared to a rmsd of 1.1 Å over 436 Cα pairs for NiV F and HeV F (Fig. 2*C*). All five disulfide bonds within a LayV F protomer are conserved compared with NiV/HeV F, suggesting they participate in proper HNV F folding. Our high-resolution LayV F structure adopts a similar architecture to a recently described LayV F structure (33) (rmsd 0.6 Å over 432 Cα pairs). The LayV F fusion peptide sequence (residues 110 to 122) is identical to MojV F and conserved with NiV/HeV F and they all exhibit an identical structure (Fig. 2 *E*–*H*). Both LayV F N-linked glycans are resolved in the cryoEM map (at positions N65 and N459), whereas NiV and HeV F harbor at least four N-linked oligosaccharides (37, 38). The LayV F N65 glycan protrudes from the trimer apex from a roughly similar location to the NiV/HeV N67 glycan (Fig. 2 *A*–*C*) which is part of an antigenic site targeted by neutralizing antibodies (21, 39). Based on the marked sequence divergence and distinct glycosylation profiles of LayV F relative to NiV and HeV F, LayV F likely has a markedly distinct antigenicity.

Postfusion LayV F forms a ~150 Å-high and ~70 Å-wide conical trimer with a prominent triple helical bundle assembled from the central helices and HR1, surrounded by three antiparallel HR2 helices yielding a 6-helix bundle at one end of the trimer. The opposite end of the molecule forms a triangular base (Fig. 2 *J*–*L*). HR1 and HR2 motifs of each F protomer interact exclusively with the other two protomers forming an intertwined network (Fig. 2 *J*–*L*). Glycans at positions N65 and N459 are resolved at the periphery of the elongated trimer (Fig. 2 *K* and *L*). In contrast to prefusion F, the fusion peptide and transmembrane regions would be located at the same end of the structure to promote membrane fusion. Postfusion LayV F shares its overall topology with other paramyxovirus postfusion F trimers as well as with postfusion coronavirus S trimers (*SI Appendix*, Fig. S4), highlighting the evolutionary relationships of these fusion machineries (40–45).

Although F refolding affects a large fraction of the trimer, the N-terminus, the β-rich domains comprising residues 281 to 420, and the upstream helix remain mostly unchanged, besides modification of their relative orientations (Fig. 2 *D* and *L*). This conformational transition leads to a greater than twofold enhancement of the surface area buried at the interface between each pair of protomers within the prefusion F state (~2,180 Å^2^) and the postfusion F state (~5,220 Å^2^), underscoring the irreversible nature of these structural rearrangements (46).

## A Generalizable Prefusion-Stabilization Strategy for HNV F Glycoproteins

**Fig. 3.**
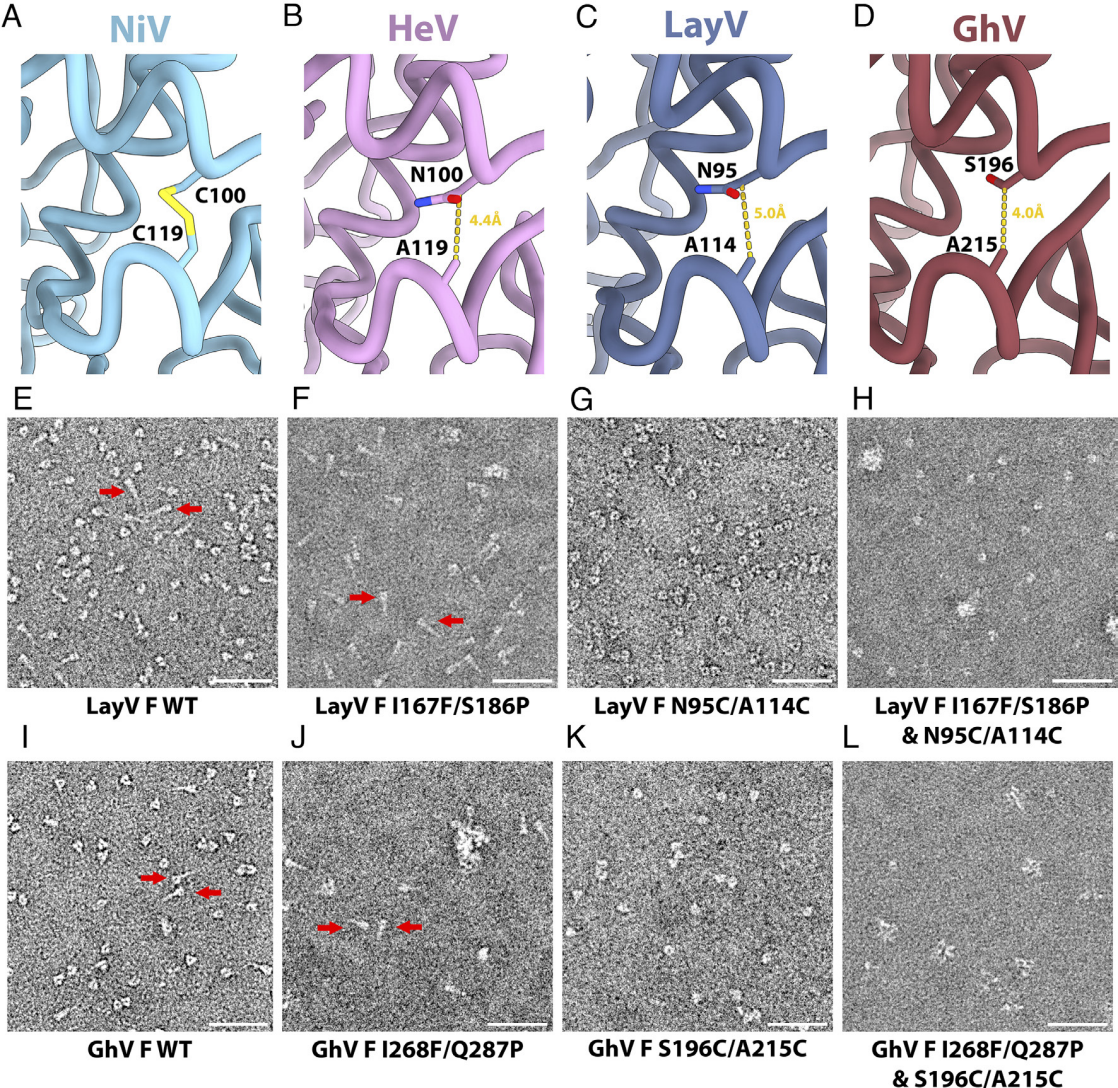
A generalizable prefusion-stabilization strategy for HNV F glycoproteins. (*A*–*D*) Zoomed-in views of NiV (PDB 6TYS) (*A*), HeV (PDB 7KI6) (*B*), LayV (*C*), and GhV (*D*) F structures. Yellow dashed lines show the distance between Cβ atoms of the labeled residues (corresponding to the mutated positions) suggesting compatibility with introduction of a disulfide bond for prefusion stabilization. (*E*–*H*) EM images of negatively stained, purified wildtype (*E*), I167F/S186P (*F*), N95C/A114C (*G*) and I167F/S186P/N95C/A114C (*H*) LayV F 2 d after purification. (*I*–*L*) EM images of negatively stained, purified wildtype (*I*), I268F/Q287P (*J*), S196C/A215C (*K*), and I268F/Q287P/S196C/A215C (*L*) GhV F 2 d after purification. (Scale bars: 50 nm.) Red arrows: postfusion F.

Spontaneous LayV F refolding underscores its metastability, typical of viral fusion proteins (40–42, 45–50). Immunization with prefusion NiV F or HeV F, but not postfusion F, elicited neutralizing antibodies (46, 51), which led us to evaluate the portability of NiV/HeV F prefusion-stabilizing mutations to LayV F. We evaluated i) the NiV L172F (cavity-filling, LayV F I167F) and S191P (postfusion central helix breaker, LayV F S186P) substitutions (51) and ii) the engineered disulfide bond spanning the F_2_ and F_1_ subunits (NiV/HeV F N100C/A119C corresponding to LayV F N95C/A114C), near the proteolytic F cleavage site (12, 22), which appear compatible with LayV F (Fig. 3 *A*–*C*). LayV F I167F/S186P mostly yielded postfusion trimers (Fig. 3 *E* and *F*) whereas LayV F N95C/A114C enabled production of well-folded prefusion F trimers (Fig. 3*G* and *SI Appendix*, Fig. S2). Combining all four mutations, however, produced prefusion F trimers and some aggregates (Fig. 3*H*). The engineered disulfide bond stapling the F_2_ and F_1_ subunits successfully reduced the LayV F metastability, stabilized prefusion F and even rescued constructs yielding postfusion F.

We decided to investigate the portability of this disulfide bond to GhV F, which shares 55% and 47% amino acid sequence identity with NiV F and LayV F, respectively. To assist the design, we determined a cryoEM structure of prefusion GhV F at 2.9 Å resolution revealing a conserved overall architecture and suggesting that the equivalent S196C/A215C disulfide would be compatible (Fig. 3 *A*–*D* and *SI Appendix*, Fig. S5 and Table 1). The I268F/Q287P and I268F/Q287P/S196C/A215C GhV F mutants mostly yielded aggregated and misfolded protein with a small fraction of postfusion trimers for I268F/Q287P and a small fraction of prefusion trimers for I268F/Q287P/S196C/A215C (Fig. 3 *J*–*L*). Similar to LayV F, the GhV S196C/A215C F disulfide mutant led to enhanced expression of prefusion F, relative to wildtype GhV F, underscoring that this strategy is broadly generalizable to HNV F glycoproteins (*SI Appendix*, Figs. S1 and S2).

## Identification of a LayV F Cross-Reactive Monoclonal Antibody

**Fig. 4.**
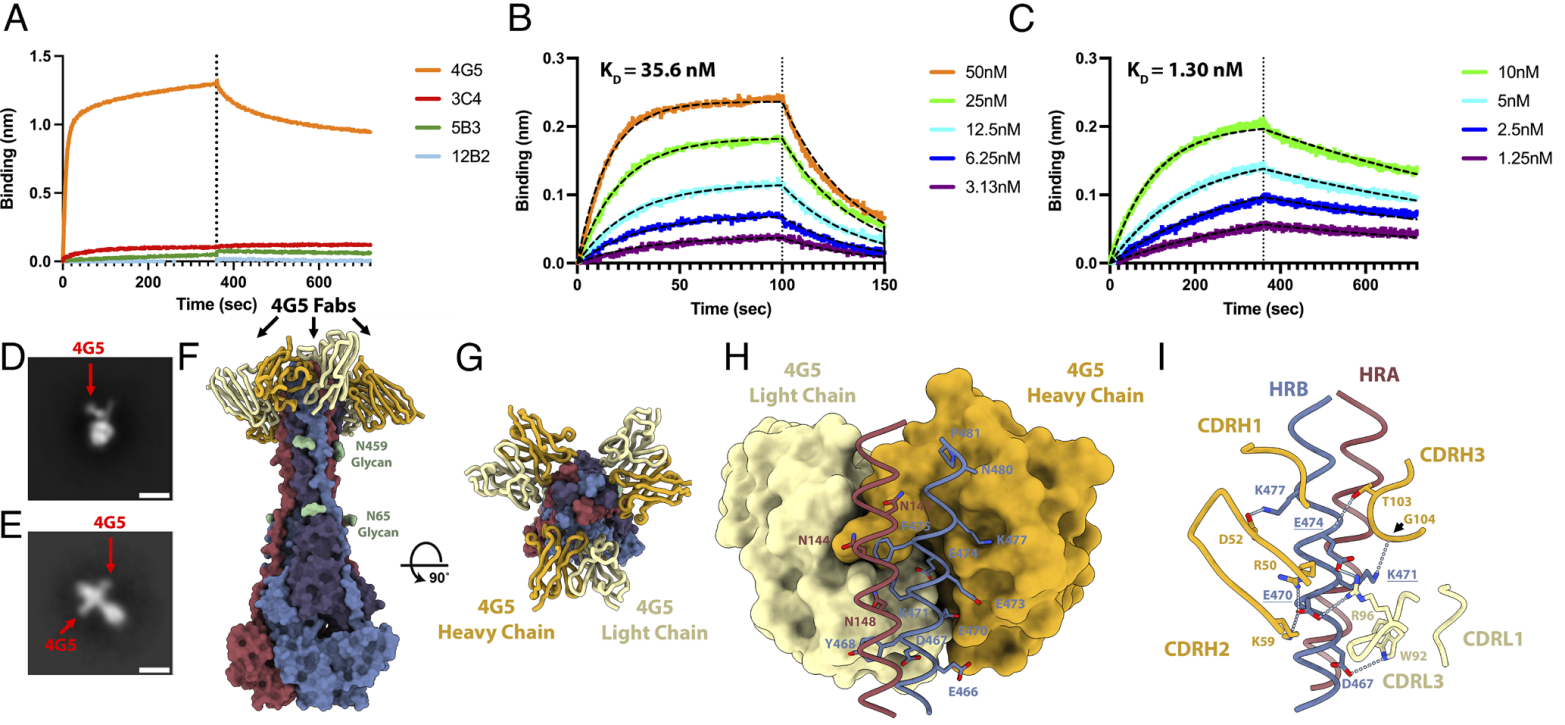
Identification of a LayV F cross-reactive mAb. (*A*) Evaluation of binding of F-directed mAbs at a concentration of 200 nM to prefusion LayV N95C/A114C F immobilized at the surface of BLI HIS1K biosensors. (*B* and *C*) Evaluation of 4G5 Fab binding kinetics and affinity to prefusion LayV N95C/A114C F (*B*) and postfusion (spontaneous refolded) wildtype LayV F (*C*) immobilized at the surface of BLI HIS1K biosensors. Color key: 4G5 Fab concentrations; black dashed lines: fit. (*D* and *E*) Two dimensional EM class averages of negatively stained 4G5 bound to wildtype prefusion (*D*) and postfusion LayV F (*E*). (Scale bar: 10 nm.) (*F* and *G*) 4G5-bound postfusion wildtype LayV F structure shown in two orthogonal orientations with each protomer colored distinctly. 4G5 Fab heavy and light chains are rendered gold and yellow, respectively. (*H* and *I*) Zoomed-in view of the interactions between the 4G5 Fab and LayV F HRA of one protomer (red) and HRB of the neighboring protomer (blue). Dashed lines indicate selected hydrogen bonds or salt bridges. Key epitope residue mutations between LayV F and NiV/HeV F are underlined.

F-directed mAbs potently neutralize NiV and HeV and protect against lethal challenge in ferrets (21–23, 52). We therefore evaluated the cross-reactivity with LayV F of NiV/HeV F-directed neutralizing mAbs using biolayer interferometry (BLI). Neither 5B3 (22) nor 12B2 (21) IgGs bound to LayV F (Fig. 4*A*), likely due to structural differences (*SI Appendix*, Fig. S6). However, 4G5 IgG, but not 3C4 IgG, bound LayV F (Fig. 4*A*), two mAbs elicited through immunizations of mice with MojV F (36). These results emphasize the close evolutionary and antigenic relationships between LayV F and MojV F, which share 90% amino acid sequence identity, and their distance with the rest of the HNV genus.

To determine whether 4G5 binding was specific for a given F conformation, we assessed binding to prefusion LayV F (N95C/A114C) and to postfusion F (obtained through spontaneous refolding) (Fig. 3*H* and *SI Appendix*, Fig. S2). 4G5 Fab recognizes prefusion and postfusion F with affinities of 35.6 and 1.30 nM, respectively (Fig. 4 *B* and *C*). EM analysis of negatively stained samples revealed binding of one 4G5 Fab to prefusion F and of three Fabs to postfusion F (Fig. 4 *D* and *E*).

To understand the molecular basis of 4G5 binding to LayV F, we determined a cryoEM structure of 4G5 Fab-bound postfusion F at 3.5 Å resolution (Fig. 4 *F* and *G* and *SI Appendix*, Fig. S7). Local refinements of the 4G5-bound membrane-proximal region and of the membrane-distal base yielded reconstructions at 3.4 and 3.2 Å, respectively (Fig. 4 *F* and *G* and *SI Appendix*, Fig. S7 and Table 1). 4G5 binds to an epitope located in the membrane proximal region of postfusion F forming an HRA/HRB six-helix bundle. Both heavy and light chains contact HRA and HRB burying a surface of ~700 Å^2^ at the interface with F using hydrogen bonding, salt bridges, and shape complementarity (Fig. 4 *H* and *I*). The 4G5 epitope comprises HRA N141, N144, and N148 from one protomer and HRB E466, D467, Y468, E470, K471, E473, E474, F475, K477, G478, and N480 from a neighboring protomer. All these residues are strictly conserved between LayV and MojV F except for the N144S substitution, rationalizing the 4G5 cross-reactivity. Epitope substitutions, including the E470_LayV_/K475_NiV_, K471_LayV_/E476_NiV_, and E474_LayV_/R479_NiV,_ likely explain the lack of 4G5 binding to NiV F (Fig. 4*I* and *SI Appendix*, Fig. S8*A*). The structure of the HRA epitope moiety is partially rearranged whereas that of the HRB epitope moiety remains identical during the prefusion to postfusion F transition. However, these two regions are adjacent to each other in postfusion F but are spatially distant in prefusion F (Fig. 2), explaining that 4G5 bound to the prefusion F (HRB) stem, which accounts for a much larger fraction of contacts formed with 4G5 than HRA, and that the affinity of 4G5 is greater for postfusion relative to prefusion F. Although 4G5 binds to both prefusion and postfusion F conformations, the antibody did not interfere with cell–cell fusion mediated by either LayV F/G or MojV F/G (*SI Appendix*, Fig. S8 *B* and *C*). 4G5 is thus unlikely to be neutralizing although it could possibly trigger Fc-mediated effector functions as observed with polyclonal antibodies in NiV-infected hamsters (53).

## Computational Design of a Stabilized LayV G Tetramer

**Fig. 5.**
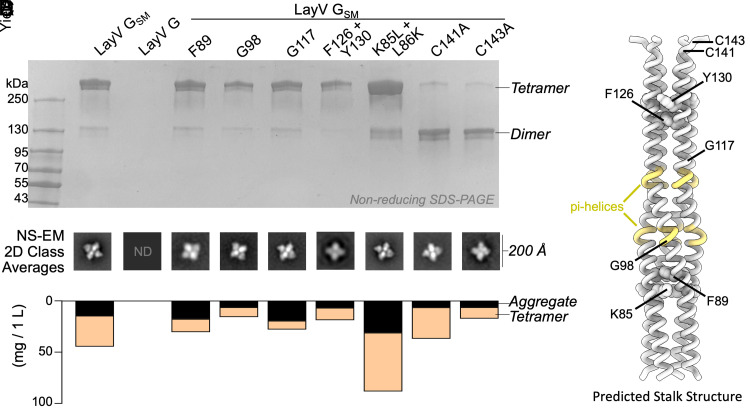
Computational design of a stabilized LayV G tetramer. (*A*) Nonreducing SDS-PAGE analysis of purified LayV G mutants after affinity purification, normalized to a standard volume to show the relative purification yield. (*B*) AlphaFold2 structure prediction of the LayV G tetrameric stalk. Residues with predicted pi-helical secondary structure are shown in yellow. (*C*) 2D class averages showing that all negatively stained (NS-EM) mutants could form tetramers similar to LayV G_SM_. (*D*) LayV G purification yield stratified by tetramers or soluble aggregates, as determined by size-exclusion chromatography.

NiV and HeV G are the targets of several potent neutralizing mAbs and the main focus of vaccine design against these pathogens. To study LayV G, we recombinantly produced the LayV G ectodomain. However, we could not detect expression of the wildtype LayV G ectodomain lacking the transmembrane and cytoplasmic domains (Fig. 5*A*).

To overcome this challenge and improve LayV G expression and stability, we set out to design G stabilizing mutations (Fig. 5). Tetrameric structure prediction of the LayV G central stalk obtained with AlphaFold2 (54) was overall consistent with the NiV G stalk organization (15) and suggested that substitutions of three aromatic residues (F89, F126, and Y130) could improve core packing (Fig. 5*B*). Furthermore, two glycine residues (G98 and G117) were anticipated to weaken the stalk secondary structure (Fig. 5*B*). Computational design of optimal residue replacements were aided by ProteinMPNN (55) and visually inspected to generate LayV G_SM_, comprising the F89L, G98T, G117A, F126V, and Y130L substitutions. Transient transfection of Expi293 cells with the LayV G_SM_ construct yielded 30 mg of purified protein per liter of culture forming well-folded tetramers with four head domains clustered around a central stalk, as visualized by negative staining EM (Fig. 5 and *SI Appendix*, Fig. S9). LayV G_SM_ harboring the L89F, T98G, A117G, or V126F/L130Y reversions had 30 to 60% reduced yields (Fig. 5*D*) indicating that the designed mutations act collectively to stabilize the ectodomain tetramer.

## Architecture of LayV G

**Fig. 6.**
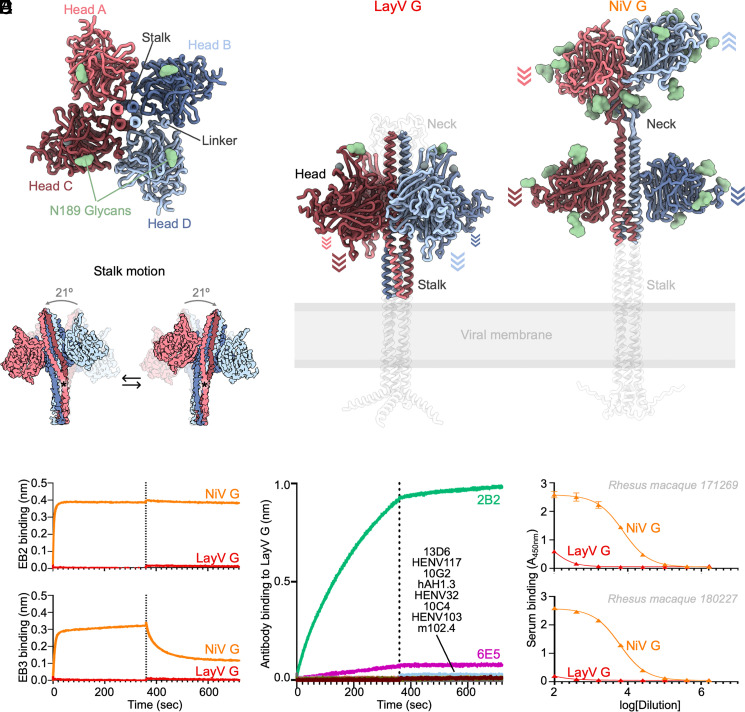
Architecture of the LayV G attachment protein. (*A*) Ribbon diagram of the LayV G ectodomain tetramer viewed along the stalk. (*B*) LayV G (*Left*) and NiV G (*Right*, PDB 7TXZ/PDB 7TY0) ectodomain structures viewed normal to the stalk. AlphaFold2 predicted structures of the stalk, neck, transmembrane, and cytoplasmic domains are shown as transparent white ribbons (as they were not empirically determined). Arrows indicate the orientation of each head domain. (*C*) Conformational flexibility of LayV G around its central stalk region revealed through cryoSPARC 3DFlex analysis of our cryoEM dataset. Residue K85 is marked with an asterisk. (*D*) Binding evaluation of 200 nM human ephrin B2 (EB2-Fc, top) and ephrin B3 (EB3-Fc, bottom) to LayV G (red) or NiV G (orange) immobilized at the surface of HIS1K BLI biosensors. (*E*) Evaluation of binding of G-directed mAbs at a concentration of 200 nM to LayV G immobilized at the surface of HIS1K BLI biosensors. (*F*) Dose–response curves for binding of NiV-M/NiV-B-elicited rhesus macaque polyclonal serum antibodies to LayV G or NiV G using ELISA.

To unveil the 3D organization of LayV G, we determined a 3.2 Å resolution cryoEM structure of LayV G_SM_ fused to a designed tetramerization motif (designated oP4h) at its N-terminus (Fig. 6 *A* and *B* and *SI Appendix*, Fig. S10 and Table 1). LayV G folds as a 150 Å-high and 140 Å-wide fourfold symmetrical tetramer with a central stalk surrounded by four β-propeller head domains (Fig. 6 *A* and *B*). The stalk region built in the cryoEM density comprises residues 68 to 142 and the β-propeller head domain comprises residues 176 to 607. Residues 166 to 175 of the intervening linker region mediates interprotomer interactions with a neighboring head domain (Fig. 6*A*). The neck region approximately spanning residues 143 to 165 was not visible in the map and could not be modeled. The N189 glycan, but not the N619 glycan, was resolved whereas the N61 and N64 glycans were not included in the construct.

The four LayV G head domains form a fourfold symmetrical clamp around the central stalk with each β-propeller interacting with two neighboring heads and with the stalk helices from two distinct protomers (Fig. 6 *A* and *B*). The connectivity between the stalk and heads is not resolved, due to the neck disorder, so each head was modeled as the same chain as the stalk it primarily interacts with [by analogy with NiV G (15)]. The LayV G head domain conformation observed in our structure differs from that found in NiV G, as the latter glycoprotein adopts an asymmetric organization of its head domains with only two of them interacting with the stalk (Fig. 6*B*). The orientation of the four LayV G heads is roughly reminiscent of the two NiV G heads positioned against the stalk, i.e. with the NiV G ephrin B2-binding site pointing toward the viral membrane, although the exact head domain positioning relative to the stalk differs. The observed LayV G head conformation and symmetry are unique among characterized paramyxovirus attachment glycoproteins (*SI Appendix*, Fig. S11). It might correspond to a snapshot of a conformation accessible to all HNV G glycoproteins [although it is distinct from that observed for NiV G (15)] or it is also possible that the yet unknown LayV G receptor imposes distinct structural constraints on G.

Although LayV G_SM_ mainly migrates as a tetramer when analyzed by nonreducing SDS-PAGE, purification of LayV G_SM_ harboring the C141A or the C143A neck residue mutations led to purification of covalent dimers (Fig. 5*A*). These data indicate that these two cysteine residues separately promote covalent LayV G dimerization and that LayV G is a dimer of dimers, similar to NiV/HeV G (56) albeit with distinct neck organizations. The stalk is a four-helix coiled coil with intervening pi-helices at residues 97 to 101 and 111 to 115 mediated by prolines at residues 102 and 116, consistent with the NiV G stalk (Fig. 5*B* and *SI Appendix*, Fig. S12). Approximately twenty additional residues could be resolved at the N-terminal region of the stalk, relative to the NiV G structure, revealing a 3_10_-helix at residues 86 to 89. CryoSPARC 3DFlex analysis (57) reveals this 3_10_-helix creates a hinge-like pivot point permitting a ~20° rotation of the membrane-proximal region relative to the head-interacting region of the stalk (Fig. 6*C* and Movie S1) that could influence receptor-binding.

Formation of the 3_10_-helix results from K85 being oriented away from and L86 toward the core of the coiled coil, positioning them in hydrophilic and hydrophobic environments, respectively (*SI Appendix*, Fig. S12). To maintain α-helical integrity, K85 would need to orient toward and L86 away from the core of the coiled coil. The equivalent residues are conserved in MojV G, GAKV G, and DARV G whereas they are respectively hydrophobic and hydrophilic in distant HNVs (*SI Appendix*, Fig. S12). Therefore, the K85L/L86K mutations are expected to favor α-helical propensity in the LayV subgroup and LayV K85L/L86K G_SM_ had a twofold enhanced production yield relative to LayV G_SM_ (Fig. 5*D*).

To further our understanding of the role of the K85L/L86K mutations on expression enhancement and rule out a possible impact of the oP4h tetramerization motif on the overall LayV G conformation, we determined a 3.2 Å resolution structure of the LayV K85L/L86K G_SM_ without oP4h (*SI Appendix*, Fig. S13). The structure is virtually indistinguishable from LayV G_SM_ with oP4h except for residues 82 to 89 which adopt a canonical α-helical structure instead of the 3_10_-helix (*SI Appendix*, Fig. S14). These findings confirm that the designed K85L/L86K mutations restore the continuity of the α-helical stalk in this region, possibly explaining enhanced expression, and show that the positioning of the head domains relative to the stalk is most likely an intrinsic property of LayV G, which does not seem to be affected by stalk modifications.

## LayV G Is Functionally and Antigenically Distinct from NiV G

Due to the close evolutionary relationship between LayV G and MojV G along with their marked divergence from NiV G and HeV G, we postulated that LayV G would not engage ephrin B2 or B3 [the NiV/HeV host receptors (16, 18)]. Indeed, we could not detect binding of human ephrin B2 or B3 to LayV G using BLI (Fig. 6*D*). Furthermore, a panel of NiV/HeV G-directed neutralizing mAbs (m102.4, HENV-32, HENV-103, HENV-117, nAH1.3), a NiV G-specific mAb (13D6) and a HeV G-specific mAb (hAH1.3) also failed to recognize LayV G (Fig. 6*E*). However, we found that one (2B2) out of three MojV G-elicited mAbs cross-reacted with LayV G (Fig. 6*E*). 2B2 did not interfere with cell–cell fusion mediated by either LayV F/G or MojV F/G (*SI Appendix*, Fig. S8 *B* and *C*) and is unlikely to be neutralizing. Collectively, these data suggest that LayV G has a different receptor tropism from NiV/HeV and distinct antigenicity.

We thus hypothesized that NiV/HeV G-elicited antibodies might not be cross-reactive with LayV G. Indeed, we could not detect appreciable antibody binding to LayV G by ELISA (enzyme-linked immunosorbent assay) using sera from two rhesus macaques vaccinated with NiV G (15) (Fig. 6*F*). These findings indicate that vaccine candidates developed against NiV/HeV would not elicit neutralizing antibodies against LayV G.

## Discussion

We reveal the ultrastructural organization of LayV F and G and provide blueprints explaining their distinct antigenicity relative to NiV/HeV F and G. The LayV G conformation observed, with all four head domains clamped down along the stalk may represent snapshots of the conformational changes leading to activation of F for membrane fusion and initiation of infection.

m102.4 is the sole mAb evaluated in the clinic against any HNV (58) and we showed here that it failed to bind to LayV G due to low sequence conservation with NiV G (*SI Appendix*, Fig. S1). Moreover, a vaccine based on the HeV G tetrameric ectodomain has been shown to protect against both NiV and HeV challenge in preclinical studies and a similar immunogen recently entered phase 1 clinical trials (NCT04199169) (26, 28, 59–63). We show that NiV G vaccine-elicited antibodies, that are neutralizing NiV, HeV, and HeV-g2 (15, 52), did not appreciably cross-react with LayV G. Countermeasures currently advanced to the clinic will thus be ineffective against LayV, urging for the development of vaccines and mAb therapies against LayV and closely related HNVs.

Structure-guided viral glycoprotein engineering has revolutionized vaccine design. Stabilization of the respiratory syncytial virus fusion glycoprotein in its prefusion conformation (DS-Cav1) propelled the development of safe and effective vaccines against this pathogen (64–66). Prefusion-stabilization (2P) of coronavirus spike glycoproteins also led to the groundbreaking development of COVID-19 vaccines in a record time (49, 67–69). We describe here the computational design of LayV G constructs, enabling high-yield recombinant production of the LayV G ectodomain tetramer, and show that a disulfide stapling HNV F trimers in the prefusion state (12, 22) is a broadly applicable F prefusion-stabilization, enabling future preclinical evaluation of vaccine candidates against LayV.

## Methods

### Cell Lines.

Expi293F was grown in Expi293 Expression Medium (Thermo Fisher) at 37 °C with 8% CO_2_ and 130 rpm. CHO-K1 cells (ATCC CCL-61) were maintained in Ham’s F-12K (Kaighn’s) Medium (Gibco) with 1% penicillin–streptomycin and 10% CCS at 37 °C with 5% CO_2_. HEK293T cells and Neuro-2a cells (ATCC CCL-131) were maintained in DMEM supplemented with 2 mM L-glutamine, 1% penicillin–streptomycin (Quality Biologicals), and 10% CCS. HEK293T cells were maintained at 37 °C with 5% CO_2_, while Neuro-2a cells were maintained at 37 °C with 8% CO_2_.

### Recombinant LayV and GhV F Ectodomain Production.

LayV F ectodomain constructs include codon-optimized LayV F (residue 1 to 482) fused to a C-terminal GCN4 followed by a linker (GSGGGS) and a hexa-histidine tag (HHHHHH). The GhV F ectodomain construct used for cryoEM includes residues 1 to 586 C-terminally fused to the I53-50A component via an intervening 16 GS linker (70) followed by a GSGGGS linker and a 6×His tag whereas the other constructs have a C-terminal GSGGGS linker followed by a 6×His tag. The LayV F wildtype, N95C/A114C, I167F/S186P, I167F/S186P/N95C/A114C and GhV F wildtype, S196C/A215C, I268F/Q287P, I268F/Q287P/S196C/A215C were synthesized by Twist Bioscience and cloned into pTwist CMV vector. LayV F and GhV F ectodomains were produced in 100 mL Expi293F cells grown in suspension using Expi293 Expression Medium at 37 °C in a humidified 8% CO_2_ incubator rotating at 130 rpm. The cultures were transfected using ExpiFectamine™ 293 Transfection Kit (ThermoFisher Scientific) with cells grown to a density of 3 million cells per mL and cultivated for 4 d. The supernatants were harvested, and proteins were purified from clarified supernatants using a 1 mL HisTrap HP column (Cytiva), buffer exchanged, concentrated, and flash frozen in either TBS (50 mM Tris, 150 mM NaCl and 10 mM EDTA at pH 8.0). SDS-PAGE was run to check purity.

### Recombinant LayV G Ectodomain Production.

LayV G ectodomains were produced in 25 mL culture of Expi293F cells. Cells grown to a density of 3 million cells per mL were transfected with the ExpiFectamine 293 Transfection Kit and cultivated for 4 d at which point the supernatant was harvested. LayV G was purified from clarified supernatants using 2 mL of cobalt resin (Takara Bio TALON), washing with 200 column volumes of 50 mM Tris-HCl pH 8.0, 150 mM NaCl, and 5 mM imidazole, and eluted with Tris-HCl pH 8.0, 150 mM NaCl, and 600 mM imidazole. Eluted protein was further purified by size exclusion chromatography using a Superose 6 increase 10/300 GL column (Cytiva) equilibrated in a buffer containing Tris-HCl pH 8.0 and 150 mM NaCl. Purified protein was concentrated using a 100 kDa centrifugal filter (Amicon Ultra 0.5 mL centrifugal filters, MilliporeSigma) to 2 mg/mL and flash frozen with liquid nitrogen. The LayV G_SM_ ectodomain has a human azurocidin signal peptide (MTRLTVLALLAGLLASSRA) followed by an N-terminal His-tag (SGPHHHHHHHHGSSP) followed by residues 72 through 624 of LayV G. The LayV G_SM_ ectodomain also has F89L, G98T, G117A, F126V, and Y130L mutations, unless otherwise denoted in the text. The LayV G_SM_ ectodomain used for structure determination also had a designed tetramerization sequence (QAEELKAIKEELKAIKEELKAIKEELKAI) between the His-tag and residue 72 of LayV G for further stabilization; though this was subsequently found to be unnecessary as LayV G_SM_ is stable on its own.

### MojV F and G-Reactive Antibody Generation.

Codon-optimized MojV (NC 025352.1) F and G open reading frames (ORFs) were synthesized by GenScript® (Piscataway, NJ, USA) and subcloned into a pcDNA3.1 Hygro CMV expression vector. The transmembrane domain (486 to 520) and C-terminal cytoplasmic tail (521 to 545) of MojV F were replaced with a GCN4 trimerization motif (GCNt) (MKQIEDKIEEILSKIYHIENEIARIKKLIGE), followed by a factor Xa protease cleavage site (IEGR) and an S-tag to generate a trimeric soluble MojV sF construct. To produce tetrameric soluble MojV G (MojV sG), the N-terminal cytoplasmic tail (2 to 37) and transmembrane domain (38 to 70) were replaced with an Igκ leader sequence (METDTLLLWVLLLWVPGSTGD) and an S-tag followed by a factor Xa protease cleavage site (IEGR) and a GCN4 tetramerization motif (GCNtet) (MKQIEDKLEEIESKLKKIENELARIKK).

For protein expression and purification, FreeStyle™ 293F cells were transfected with sF, and Neuro-2a cells with sG plasmid constructs. Their stable cell lines were obtained through two rounds of limiting dilutions. Stably expressing MojV sF and sG secreted into FreeStyle™ 293 Expression Medium were collected and first purified through a S-agarose affinity column (XK 26 column). For MojV sG, the column was washed with 3× bed volumes of Buffer I (PBS, 0.1% Triton® X-100, 0.3 M NaCl), followed by 6× bed volumes of Wash Buffer II (PBS, 0.1% Triton® X-100). The protein was eluted with Elution buffer (0.2 M citrate, pH 2), then neutralized with buffer (1 M Tris-HCl pH 8.0 or 1 M HEPES buffer pH 9.0). The protein was buffer exchanged to PBS, pH7.4. For MojV sF, the column was washed with 3× bed volumes of Wash buffer I (PBS, 0.5% Triton X-100, 0.5 M NaCl, 0.1 M L-Arginine, 0.02 M Tris-HCl, pH 7.5. followed by 6× bed volumes of Wash buffer II (PBS, 0.1% Triton® X-100, 0.1 M L-Arginine). The protein was eluted with Elution buffer (0.2 M citrate, 0.2 M L-Arginine, pH 2) and neutralized with buffer (1 M HEPES buffer pH 9.0). The protein was buffer exchanged into PBS, 0.01% Triton® X-100, 0.1 M L-Arginine. S-tag was removed with the Factor Xa Cleavage Capture kit (Millipore Sigma). The untagged sF or sG was further purified with a size-exclusion column (HiLoad 16/60 Superdex 200 prep grade XK 16 gel filtration column (GE Healthcare). The buffer PBS was used for sG and the PBS with 0.01% Triton X-100 was used for sF purification.

To generate F- or G-directed mAbs, purified sF or sG were used to immunize BALB/c mice purchased from Jackson Labs (Bar Harbor, ME, USA). All animal studies were carried out under an approved protocol MIC-16-262 for animal experiments obtained from the Uniformed Services University Animal Care and Use Committee. All mice were immunized 4 times.The administered antigens (sF/sG) were formulated in a Sigma adjuvant system (Sigma-Aldrich Co. LLC, St. Louis, MO). Mouse splenic lymphocytes were isolated 4 d following a final immunization without adjuvant and fused with SP2/0 cells using polyethylene glycol by standard methods. Hybridoma supernatants producing MojV F (4G5) or G(2B2) -specific antibodies were identified by ELISA using purified S-peptide-cleaved MojV sF or sG. The anti-F or G mAbs were prepared under serum-free conditions using HyClone SFM4MAb (Thermo Fisher Scientific Inc., Rockford, IL) and purified by protein G-Sepharose affinity chromatography.

### Antibody Production.

nAH1.3 heavy chain and light chain were codon optimized and synthesized by GeneArt (2019AAG4YC) with a signal sequence (MPMGSLQPLATLYLLGMLVASVLA) at the N termini of both heavy and light chains, as well as a StrepII tag (WSHPQFEK) linked by a linker sequence (GSGGGS) at the C terminal of the heavy chain. The synthesized heavy and light chain genes were each subcloned into a pcDNA3_1(+)_MAr vector. nAH1.3 IgG was produced in 25mL Expi293F cells grown in suspension using Expi293 Expression Medium (Thermo Fisher) at 37 °C in a humidified 8% CO2 incubator rotating at 130 rpm. The cultures were transfected with 1:1 ratio of heavy chain and light chain plasmids using ExpiFectamine™ 293 Transfection Kit with cells grown to a density of 3 million cells per mL and cultivated for 6 d. The supernatants were harvested, and proteins were purified from clarified supernatants using a 1mL StrepTrap™ HP column (Sigma), buffer exchanged, concentrated and flash frozen in either TBS or PBS. SDS-PAGE was run to check purity.

HENV-32, HENV-103, and HENV-117 IgG genes were codon-optimized for a mammalian cell expression system, synthesized, and cloned into a pCDNA3.1+ vector by GenScript. The HENV-32 light-chain sequence was obtained from the Fab sequence of crystal structure Protein Data Bank (PDB 6VY4, and the HENV-32 heavy-chain sequence was reconstructed by combining the Fab sequence of crystal structure PDB 6VY4 with a consensus sequence of IGHG1 (P01857). nAH1.3 and HENV-32 heavy chains include a StrepII tag (WSHPQFEK) linked by a linker sequence (GSGGGS) at the C terminus. HENV-103 and HENV-117 light- and heavy-chain constructs have been produced by replacing the variable domain of HENV-32 constructs with the corresponding HENV-103 or HENV-117 sequence. The C-terminal StrepII tag and linker have been removed for HENV-103 and HENV-117 heavy-chain constructs.

m102.4 was isolated from a recombinant human phage-displayed Fab library and prepared as previously described (71). Briefly, CHO-K1 cells were transfected with a linearized m102.4 PDR12 construct and the antibody was purified with protein A from a selected stable m102.4-producing cell line (71). The human IgG1 m102.4 mAb has been extensively characterized as a highly potent HeV and NiV cross-reactive mAb (27, 58, 71–74).

5C4, 5B3, 12B2, 6E5, 13D6, 10G2, hAH1.3 IgGs were produced from hybridomas and were previously described as refs. 36 and 46.

### BLI.

Assays were performed on an Octet Red (ForteBio) instrument at 30 °C with shaking at 1,000 RPM.

For anti-HNV mAbs screening, anti-penta His (HIS1K) biosensors were hydrated in water for 10 min prior to a 120 s incubation in 10× kinetics buffer (KB, undiluted). LayV F N95C/A114C or LayV G_SM_ was loaded at 10 μg/mL in 10× KB for 360 s prior to baseline equilibration for 300 s in 10× KB. Association of anti-HNV IgGs in 10× KB at 200 nM concentration was carried out for 360 s prior to dissociation for 360 s.

For 4G5 K_D_ determination of prefusion LayV F, HIS1K biosensors were hydrated in water for 10 min prior to a 120 s incubation in 10× KB. LayV F N95C/A114C was loaded at 20 μg/mL in 10× KB for 300 s prior to baseline equilibration for 300 s in 10× KB. Association of 4G5 Fab in 10× KB in a twofold dilution series from 100 nM to 3.125 nM was carried out for 100 s prior to dissociation for 100 s.

For 4G5 K_D_ determination of postfusion LayV F (spontaneously refolded over 4 mo at 4 °C), HIS1K biosensors were hydrated in water for 10 min prior to a 120 s incubation in 10× KB. Postfusion LayV F was loaded at 20 μg/mL in 10× KB for 300 s prior to baseline equilibration for 300 s in 10× KB. Association of 4G5 Fab in 10× KB in a twofold dilution series from 40 nM to 1.25 nM was carried out for 360 s prior to dissociation for 360 s.

For 4G5 K_D_ determination, the data were baseline subtracted prior to fitting performed using a 1:1 binding model and the ForteBio data analysis software. Mean kon, koff values were determined with a global fit applied to all data. The experiments were done with two separate purification batches and data plotted in Prism (GraphPad).

### ELISA.

First, 96-well Maxisorp plates (Thermo Fisher) were coated overnight at 4 °C with 2 µg/mL of NiV G or LayV G_SM_ in 50 mM Tris and 150 mM NaCl at pH 8. Plates were slapped dry, washed 3× in TBST, and blocked with Blocker Casein (ThermoFisher) for 1 h at 37 °C. Then, plates were slapped dry and washed 4× in TBST, and 1:4 serial dilutions of NHP sera were made in 50 μL TBST and incubated at 37 °C for 1 h. Plates were slapped dry and washed 4× in TBST followed by addition of 50 μL 1:5,000 Goat anti-Human IgG Fc Secondary Antibody with HRP (Invitrogen) for 1 h at 37 °C. Plates were slapped dry and washed 4× in TBST followed by addition of 50 μL TMB Microwell Peroxidase (Seracare). The reaction was quenched after 4 min with 1 N HCl and the A450 of each well was read using a BioTek Synergy Neo2 plate reader. Data were plotted and fit in Prism (GraphPad) using nonlinear regression sigmoidal, 4PL, X is log(concentration).

### Negative Stain EM.

Three μL of purified sample of each F or G construct was applied to glow discharged 300 mesh copper grids (Ted Pella) covered with evaporated continuous carbon for 1 min before blotting away excess liquid with Whatman no. 1 filter paper. Grids were stained twice with 3 μL of 2% (w/v) uranyl formate and imaged using an FEI Tecnai Spirit 120 kV electron microscope equipped with a Gatan Ultrascan 4000 CCD camera. The pixel size at the specimen level was 1.60 Å. Data collection was performed using Leginon (75) and processing using cryoSPARC (76).

### Membrane-Anchored LayV F and G Expression Plasmids and Peptide Synthesis.

Codon-optimized LayV F and G ORFs were synthesized by GenScript and subcloned into a mammalian promoter modified expression vector pcDNA3.1-CMV (77). To enable detection, an S peptide tag (KETAAAKFERQHMDS) was inserted at the C-terminal end of LayV F and at the N-terminal end of LayV G. A peptide corresponding to the HR2 domain of LayV F (LayV HR2, KIDIGNQLAGINQTLQNAEDYIEKSEEFLKGINPSI) and the corresponding scrambled peptide (scrambled LayV HR2, SIANIQEKDIIKLETEDPEIYAGNKLGSQILNFGQN) were synthesized by Biosynth (Gardner, MA, USA).

### Western Blot.

CHO-K1, HEK293T, and Neuro-2a cells were transfected with pcDNA3.1-LayV F, pcDNA3.1-LayV G, or pcDNA3.1-LayV F and pcDNA3.1-LayV G encoding S-tagged constructs. As a reference, cells were transfected with pcDNA3.1-MojV F, pcDNA3.1-MojV G or pcDNA3.1-MojV F and pcDNA3.1-MojV G encoding S-tagged constructs (36). At 48 h post transfection, cells were lysed with 1× radioimmunoprecipitation assay Lysis and Extraction Buffer containing a protein inhibitor cocktail and incubated with S protein agarose beads at 4 °C overnight before SDS-PAGE and detection with an anti-S-peptide-HRP conjugated antibody.

To assess the effect of the cysteine protease inhibitor E64d on HNV F cleavage, mouse Neuro-2a cells were transfected with pcDNA 3.1 bearing the C-terminally S- tagged HeV F, NiV F, LayV F, or MojV F, and incubated with 50 µM E64d or 0.5% methanol. E64d was dissolved in solvent (methanol:H_2_O = 1:1) to 5 mM and diluted in DMEM. The cells were harvested at 48 h posttransfection and the lysates were immuno-precipitated with S agarose beads before SDS-PAGE and probed with rabbit anti-S peptide:HRP conjugated antibody.

### Cell–Cell Fusion Assay.

We used a quantitative dual-split-reporter luciferase-based cell–cell fusion assay as previously described (78). Briefly, the effector cells (Neuro-2a cells) were transfected with pcDNA3.1-LayV F, or pcDNA3.1-LayV G or pcDNA3.1-LayV F and pcDNA3.1-LayV G together with one-half of the dual-split-reporter luciferase expression plasmid (DSP1–7). As a control, Neuro-2a cells were cotransfected with pcDNA3.1-MojV F, pcDNA3.1-MojV G, and DSP 1 to 7. Each target cell (HEK293T or Neuro-2a cells, endogenously expressing HNV receptors) was transfected with the other half of the dual-split reporter plasmid (DSP 8 to 11). After 48 h posttransfection, the effector cells were gently detached and applied onto the target cells in equal numbers. At 48 h post mixing, EnduRen (Live cell substrate for the luciferase) was added to the plate. Content mixing between the effector cells and the target cells as a result of cell–cell fusion was measured using a luminometer (as RLU, relative luminescence unit). For the assessment of cell–cell fusion mediated by LayV F/G or MojV F/G with LayV HR2 and scrambled LayV HR2 peptides and with the 4G5 or 2B2 IgGs, the Neuro-2a effector cells transfected with LayV F/G or MojV F/G were pretreated with peptide or IgG for 10 min at 37 °C, then gently detached and applied onto the HEK293T target cells with equal densities. The D61 (anti-HIV gp41) IgG was used as a negative control. Each assay was done for three biological replicates.

### CryoEM Sample Preparation and Data Collection.

CryoEM grids of prefusion LayV F were prepared by applying 3 μL of LayV F wildtype at 0.14 mg/mL to holey carbon grids covered with graphene oxide, prior to plunge freezing using a Vitrobot MarkIV (ThermoFisher Scientific) with a blot force of −1 and 3 s blot time at 100 % humidity and 22 °C. Data were acquired using an FEI Titan Krios operated at 300 kV and equipped with a Gatan K3 direct detector and Gatan Quantum GIF energy filter, operated in zero-loss mode with a slit width of 20 eV. The dose rate was adjusted to 15 counts/pixel/s, and each movie was fractionated in 75 frames of 40 ms. Automated data collection was carried out using Leginon at a nominal magnification of 105,000× with a pixel size of 0.843 Å and a defocus range of −0.7 to −1.7 μm (75).

CryoEM grids of postfusion LayV F were prepared by applying 3 μL of 2.7 mg/mL LayV F wildtype (spontaneous refolded to postfusion over 3 mo at 4 °C) with 10 mM OG detergent to 2.0/2.0 UltraFoil (79) grid (200 mesh) and plunge freezing using a vitrobot MarkIV using a blot force of 0 and 6 s blot time at 100% humidity and 22 °C. Data were acquired using SerialEM (80, 81) to control an FEI Glacios operated at 200 kV and equipped with a Gatan K3 Summit. The dose rate was adjusted to 7.5 counts/pixel/s, and each movie was acquired in 100 frames of 50 ms with a defocus range of −0.7 to −1.7 μm and a pixel size of 0.89 Å.

CryoEM grids of 4G5-bound LayV F were prepared by applying 3 μL of 1 mg/mL LayV F wildtype (spontaneous refolded to postfusion over 4 mo at 4 °C) with 10 mM OG detergent to 2.0/2.0 UltraFoil (79) grid (200 mesh) and plunge freezing using a vitrobot MarkIV with a blot force of 0 and 6 s blot time at 100% humidity and 22 °C. Data were acquired using an FEI Titan Krios operated at 300 kV and equipped with a Gatan K3 Summit and Gatan Quantum GIF operated in zero-loss mode with a slit width of 20 eV. The dose rate was adjusted to 15 counts/pixel/s, and each movie was fractionated in 75 frames of 40 ms. Automated data collection was carried out using SerialEM (80, 81) at a nominal magnification of 105,000× with a pixel size of 0.843 Å and a defocus range of −1.3 to −1.7 μm.

CryoEM grids of LayV G_SM_ (oP4h) were prepared by applying 3 μL of 0.6 mg/mL or 2 mg/mL of sample with 0.01% fluorinated octyl-maltoside (FOM, Anatrace) detergent to 2.0/2.0 UltraFoil (79) grid (200 mesh) and plunge freezing using a Vitrobot MarkIV with a blot force of −1 and 6 s blot time at 100% humidity and 22 °C. Data were acquired using Leginon (75) to control an FEI Glacios operated at 200 kV and equipped with a Gatan K3 Summit. The dose rate was adjusted to 7.5 counts/pixel/s, and each movie was acquired in 100 frames of 50 ms. For LayV G_SM_ with and without FOM, 4,894 and 4,086 micrographs, respectively, were collected in a single session with a defocus range of −0.5 to −2.4 μm and a pixel size of 0.89 Å.

CryoEM grids of LayV K85L/L86K G_SM_ without oP4h fusion were prepared by applying 3 µL of sample at 2 mg/mL with 0.02% FOM to 2.0/2.0 UltraFoil (79) grid (200 mesh) and plunge frozen using a Vitrobot MarkIV with a blot force of −1 and 3 s blot time at 100% humidity and 22 °C. Data were acquired using Leginon (75) to control an FEI Glacios operated at 200 kV and equipped with a Gatan K3 Summit. The dose rate was adjusted to 7.5 counts/pixel/s and each movie was acquired in 100 frames of 50 ms with a defocus range of −0.7 to −1.8 μm and a pixel size of 0.89 Å.

CryoEM grids of GhV F I53-50A were prepared by applying 3 μL of sample at 0.05 mg/mL to holey carbon grids covered with graphene oxide, prior to plunge freezing using a Vitrobot MarkIV with a blot force of −1 and 3 s blot time at 100% humidity and 22 °C. Data were acquired using an FEI Titan Krios operated at 300 kV and equipped with a Gatan K3 Summit and Gatan Quantum GIF operated in zero-loss mode with a slit width of 20 eV. The dose rate was adjusted to 15 counts/pixel/s, and each movie was fractionated in 75 frames of 40 ms. The nominal magnification was 105,000× with a pixel size of 0.843 Å and the defocus range was −0.5 to −2.5 μm.

### CryoEM Data Processing.

For the prefusion LayV F structure, movie frame alignment, estimation of the microscope contrast-transfer function (CTF) parameters, particle picking, and extraction were carried out using Warp (82). Reference-free 2D classification and nonuniform refinement with reference from previously determined HNV F were performed using cryoSPARC (76). Bayesian polishing was done in Relion (83), followed by nonuniform refinement (84) another round of reference-free 2D classification and a final nonuniform refinement using cryoSPARC.

For the postfusion LayV F structure, movie frame alignment, estimation of the microscope CTF parameters, particle picking, and extraction with 2× binning were carried out using cryoSPARC (76). Reference-free 2D classification was performed using cryoSPARC (76). Ab initio structure reconstruction was performed in cryoSPARC using three classes (76). Subset of good postfusion LayV F particles were used for homologous refinement followed by nonuniform refinement. Particles were extracted with original pixel size followed by another round of nonuniform refinement.

For the structure of LayV F postfusion in complex with 4G5 Fab, movie frame alignment, estimation of the microscope CTF parameters, particle picking, and extraction were carried out using Warp (82). Two rounds of reference-free 2D classification were performed using cryoSPARC (76). Heterogeneous refinement was used to sort good particles followed by homologous refinement and nonuniform refinement with per-particle defocus refinement in cryoSPARC (84). Selected particle images were subjected to the Bayesian polishing procedure (83) implemented in Relion before performing another round of homologous refinement and nonuniform refinement in cryoSPARC. Local nonuniform refinements were performed for the proximal and distal parts of the complex yielding reconstructions at 3.4 Å and 3.2 Å resolution, respectively.

For the LayV G_SM_ structure (oP4h), movie frame alignment and binning to 1.78 Å was carried out using Warp (82), estimation of the microscope CTF parameters, particle picking, and extraction (with a box size of 440 pixels^2^) was carried out in cryoSPARC using Topaz (76, 85). Reference-free 2D classification was performed using cryoSPARC to select well-defined particle images. Ab initio structure reconstruction, heterogeneous refinement, and nonuniform refinement in cryoSPARC were then performed. 3D classification with 50 iterations each (no angular sampling) was carried out using Relion (86) without imposing symmetry to select well-defined particle classes. Particle images were then subjected to Bayesian polishing using Relion (83) during which the box size was adjusted to 440 Å with a pixel size of 0.89 Å. Another round of nonuniform refinement in cryoSPARC was performed concomitantly with global and per-particle defocus refinement as well as beam tilt refinement (84, 86). The particles from this refinement were used with 3D Flex (57) in cryoSPARC to determine the motions of LayV G, using the default setting for Flex Data Prep, Flex Mesh Prep, Flex Train, and Flex Generate, with the exception that the training box size was set to 220 pixels.

For the structure of LayV G_SM_ harboring the K85L/L86K mutations without oP4h, movie frame alignment and binning to 1.78 Å was carried out using Warp. Estimation of the microscope CTF parameters, particle picking, and extraction (box size of 440 pixels) was carried out in cryoSPARC. Reference-free 2D classification was performed using cryoSPARC. A subset of exemplar 2D classes was used in cryoSPARC with Topaz Train to generate a model for the automatic picking of good particles. Ab initio reconstruction was performed in cryoSPARC using three classes and a subset of good particles was used for nonuniform refinement. Particle images then underwent Bayesian polishing using Relion during which the box size was adjusted to 440 Å with a pixel size of 0.89 Å. Another round of nonuniform refinement was performed alongside global contrast transfer function refinement to account for beam tilt. A final round of 2D classification and selection was performed to remove the remaining bad particles from the dataset.

For the structure of GhV F, movie frame alignment, estimation of the microscope CTF parameters, particle picking, and extraction were carried out using Warp (82). Two rounds of reference-free 2D classification were performed using cryoSPARC (76) to select well-defined particle images. 3D refinements were carried out using nonuniform refinement (84) along with per-particle defocus refinement in cryoSPARC. Selected particle images were subjected to Relion Bayesian polishing (83) before performing another round of nonuniform refinement in cryoSPARC with per-particle defocus refinement.

Reported resolutions are based on the gold-standard Fourier shell correlation (FSC) of 0.143 criterion and FSC curves were corrected for the effects of soft masking by high-resolution noise substitution (87, 88).

### CryoEM Model Building and Analysis.

Initial models of both prefusion and postfusion LayV F were built automatically using ModelAngelo (89). UCSF ChimeraX (90) and Coot (91, 92) were used for docking and manually building into the cryoEM maps. Models were refined into the cryoEM maps using Rosetta (93–95), Phenix (96), and ISOLDE (97) Validation used MolProbity (98), Phenix (96), and Privateer (99). Figures were generated using UCSF ChimeraX (90) and UCSF Chimera (100).

## Supplementary Material

Appendix 01 (PDF)

Movie S1. 3DFlex cryoEM density series oscillating between 41 frames showing movement of the LayV G_SM_ (oP4h) stalk.

## Data Availability

CryoEM data have been deposited in EMDB/PDB (listed in *SI Appendix*, Table 1).
