# Supplementary material for: Structure and design of Langya virus glycoprotein antigens
Source: Proc Natl Acad Sci U S A. 2024 Apr 9;121(16):e2314990121. doi: 10.1073/pnas.2314990121 (PMC11032465; doi:10.1073/pnas.2314990121)
Supplement: Supplementary file 1 — Appendix 01 (PDF) [file pnas.2314990121.sapp.pdf]

A

F	NiV-M	NiV-B	HeV	HeV-g2	GhV	CedV	MojV	LayV	DARV	GAKV
NiV-M		98.9	88.1	88.4	54.94	45.28	42.69	44.4	43.82	40.6
NiV-B	98.9		90.04	89.29	55.13	45.28	43.08	43.55	43.82	44.47
HeV	88.1	90.04		95.6	55.66	47.49	43.63	44.19	43.95	39.9
HeV-g2	88.4	89.29	95.6		54.7	47.38	43.23	43.6	43.21	44.44
GhV	54.94	55.13	55.66	54.7		42.01	45.49	46.53	44.29	44.38
CedV	45.28	45.28	47.49	47.38	42.01		36.06	36.53	37.87	37.55
MojV	42.69	43.08	43.63	43.23	45.49	36.06		90.35	69.79	57.91
LayV	44.4	43.55	44.19	43.6	46.53	36.53	90.35		70.86	56.83
DARV	43.82	43.82	43.95	43.21	44.29	37.87	69.79	70.86		60.58
GAKV	40.6	44.47	39.9	44.44	44.38	37.55	57.91	56.83	60.58	

B

G	NiV-M	NiV-B	HeV	HeV-g2	GhV	CedV	MojV	LayV	DARV	GAKV
NiV-M		95.68	78.74	79.33	28.06	32.51	23.28	22.2	24.22	19.9
NiV-B	95.68		78.24	78.96	26.45	32.2	22.75	22.26	23.44	20.3
HeV	78.74	78.24		92.85	28.75	30.57	22.1	23.06	25	19.77
HeV-g2	79.33	78.96	92.85		29.08	31.13	22	22.62	23.64	19.97
GhV	28.06	26.45	28.75	29.08		27.42	20.13	19.93	20.49	19.44
CedV	32.51	32.2	30.57	31.13	27.42		19.85	19.74	20.9	18.7
MojV	23.28	22.75	22.1	22	20.13	19.85		86.04	52.03	30.18
LayV	22.2	22.26	23.06	22.62	19.93	19.74	86.04		53.79	28.9
DARV	24.22	23.44	25	23.64	20.49	20.9	52.03	53.79		30.43
GAKV	19.9	20.3	19.77	19.97	19.44	18.7	30.18	28.9	30.43	

Fig S1. HNV glycoprotein sequence conservation. A-B, Pairwise amino acid sequence identity of HNV F (A) and G (B) glycoproteins. The HNV F alignment uses NiV-M (AAK50553.1), NiV-B (AEZ01396.1), HeV (AEQ38140.1), HeV-g2 (QYC64604.1), GhV (YP_009091837.1), CedV (YP_009094085.1), LayV (UUV47205.1), MojV (YP_009094094.1), DARV (QYO90531.1) and GAKV (QYO90517.1). The HNV G alignment uses NiV-M (NP_112027.1), NiV-B (AAY43916.1), HeV (NP_047112.2), HeV-g2 (QYC64605.1), GhV (YP_009091838.1), CedV (YP_009094086.1), LayV (UUV47206.1), MojV (YP_009094095.1), DARV (QYO90532.1) and GAKV (QYO90518.1). Cells are colored based on amino acid sequence identity, where blue indicates high sequence identity and red indicates low sequence identity.

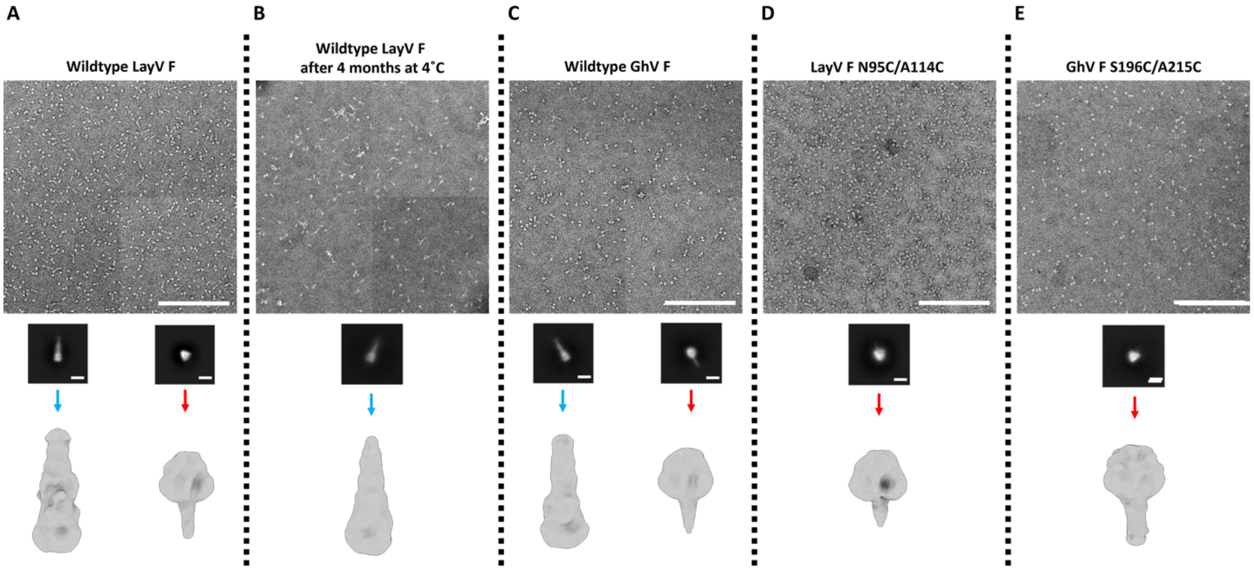

Fig. S2. EM data processing of negatively stained HNV F glycoproteins. A-E, Representative micrograph, 2D class averages and 3D reconstructions are shown for negatively stained wildtype LayV F (A), wildtype LayV F spontaneous refolded to the postfusion state (B), wildtype GhV F (C), LayV F N95C/A114C (D) and GhV F S196C/A215C (E). The scale bar represents 200 nm for micrographs or 100 Å for 2D class averages.

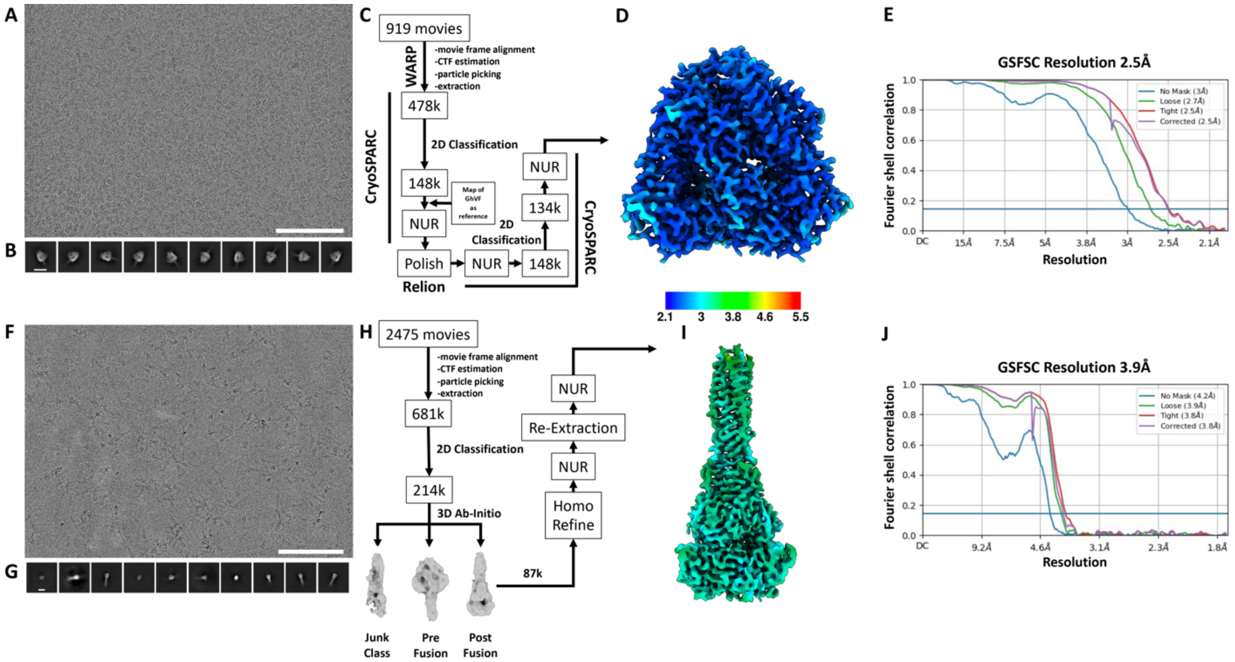

Fig. S3. CryoEM data processing for wildtype prefusion and postfusion LayV F datasets. A-B, Representative electron micrograph (A) and 2D class averages (B) of

prefusion LayV F embedded in vitreous ice. The scale bar represents 100 nm (A) or 100 Å (B). **C,** CryoEM data processing flow chart for prefusion LayV F. CTF: contrast transfer function; NUR: non-uniform refinement; Polish: Bayesian particle polishing implemented in Relion. **D,** Prefusion LayV F map colored by local resolution computed using cryoSPARC. **E,** Fourier shell correlation curves with the 0.143 cutoff used indicated with an horizontal blue line. **F-G,** Representative electron micrograph (F) and 2D class averages (G) of postfusion LayV F. The scale bar represents 100 nm (F) or 100 Å (G). **H,** CryoEM data processing flow chart for postfusion LayV F using Cryosparc. NUR: non-uniform refinement; Homo Refine: homogeneous refinement. **I,** Postfusion LayV F map colored by local resolution computed using cryoSPARC. **J,** Fourier shell correlation curves with the 0.143 cutoff used indicated with an horizontal blue line.

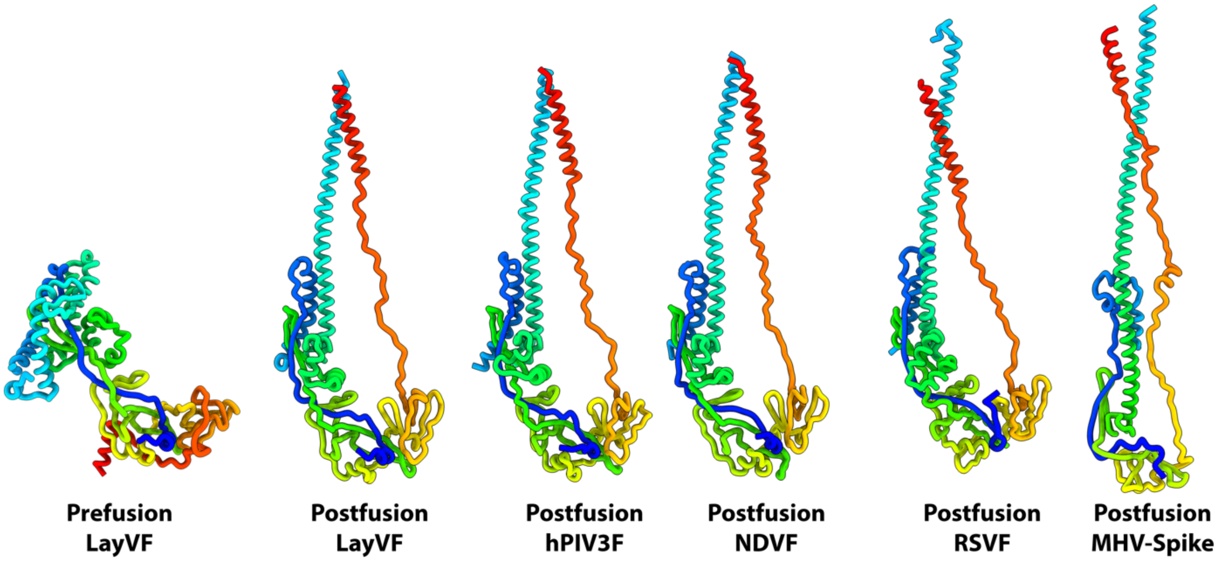

Fig. S4 Conservation of the general architecture of paramyxovirus and coronavirus fusion proteins.

Ribbon diagrams underscoring the architectural conservation among paramyxovirus, respirovirus and coronavirus postfusion structures. All models are colored using a rainbow scheme from blue (N-terminus) to red (C-terminus). The structures rendered are human parainfluenza virus 3 F (hPIV3 F, PDB 1ZTM(46)); Newcastle disease virus F (NDV F, PDB 3MAW(101)); respiratory syncytial virus F (RSV F, PDB 3RRR(43)); mouse hepatitis virus spike (MHV, PDB 6B3O(42)).

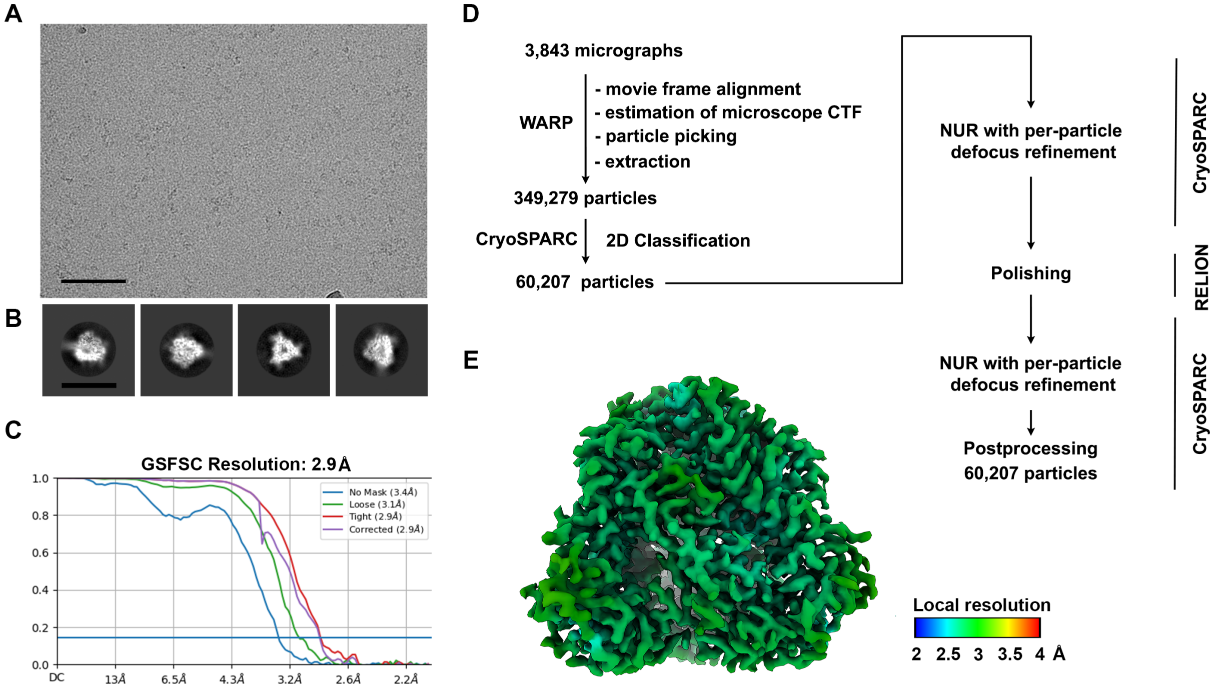

Fig. S5 CryoEM data processing pipeline for GhV F. A-B, Representative electron micrograph (A) and 2D class averages (B) of GhV F embedded in vitreous ice. The scale bar represents 100 nm (A) or 150 Å (B). **C,** Gold-standard Fourier shell correlation curve for the GhV F reconstruction. The 0.143 cutoff is indicated with an horizontal blue line. **D,** Data processing flowchart. CTF: contrast transfer function; NUR: non-uniform refinement; Polishing: Bayesian particle polishing implemented in Relion. **E,** Sharpened GhV F reconstruction colored according to local resolution calculated using cryoSPARC.

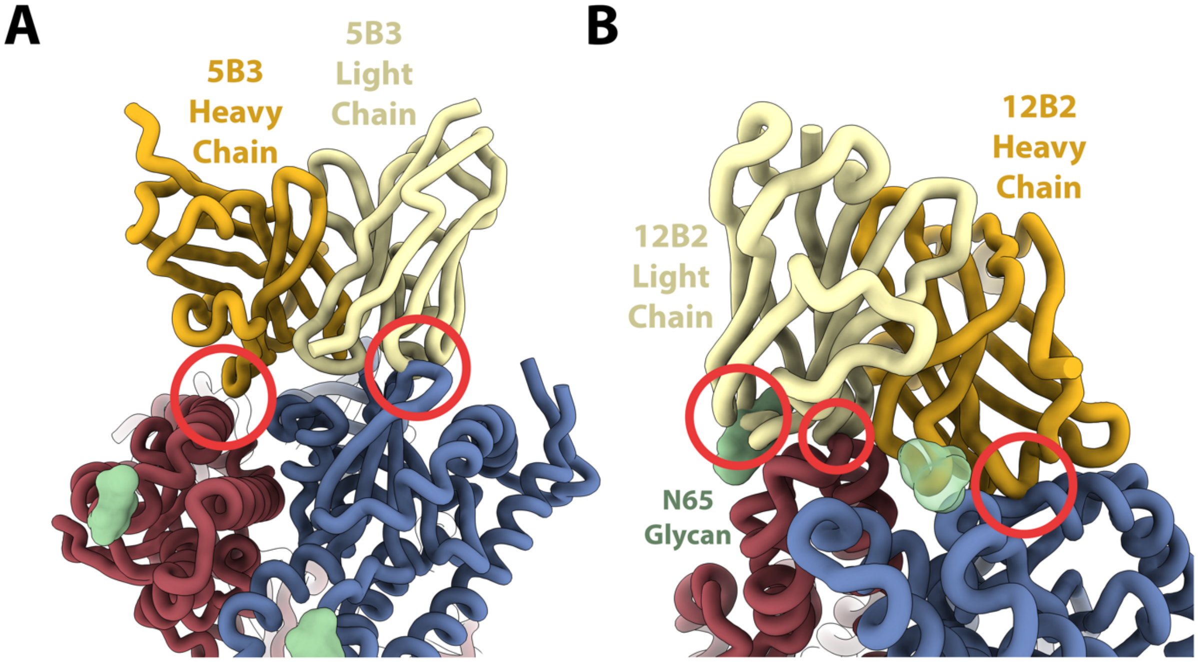

Fig. S6 Incompatibility of the prefusion LayV F structure with binding of two NiV/HeV F neutralizing mAbs. A, Superimposition of the 5B3-bound NiV F structure (PDB 6TYS(18)) onto prefusion LayV F. Two LayV F protomers are shown in blue and red whereas the 5B3 heavy and light chains are rendered gold and yellow respectively. NiV F is omitted for clarity. N-linked glycans are shown as green surfaces. Red circles indicate potential clashes. **B,** Superimposition of the 12B2-bound HeV structure (PDB 7KI4(17)) onto prefusion LayV F with the same color and representation scheme as in panel A.

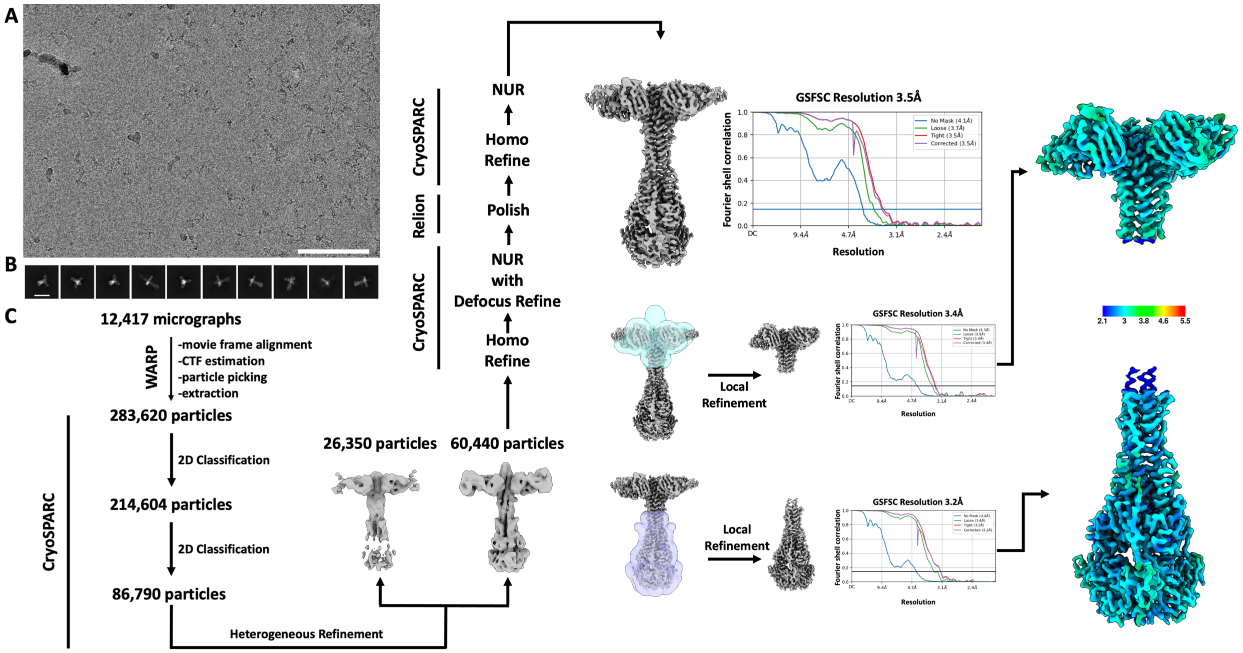

Fig. S7 CryoEM data processing pipeline for postfusion LayV F in complex with the 4G5 Fab. A-B, Representative electron micrograph (A) and 2D class averages (B) of 4G5-bound postfusion LayV F embedded in vitreous ice. The scale bar represents 100 nm (A) or 200 Å (B). **C,** CryoEM data processing flow chart including local resolution maps computed using cryoSPARC. The masks used for local refinement are shown in pink and purple. Homo Refine: homogeneous refinement. CTF: contrast transfer function; NUR: non-uniform refinement; Polish: Bayesian particle polishing implemented in Relion.

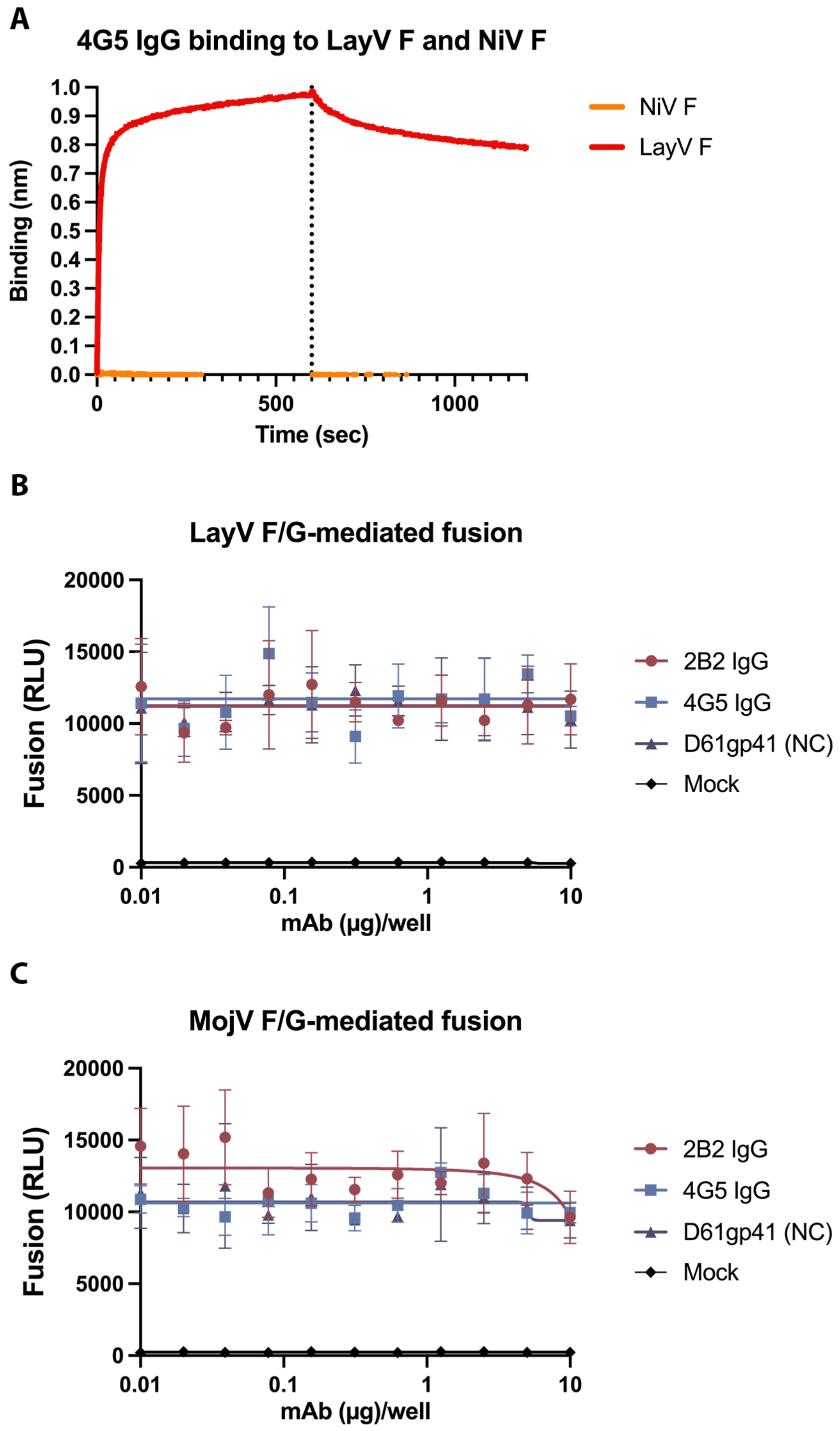

Fig. S8 Characterization of MojV and LayV F/G cross-reactive antibodies. A, Biolayer interferometry binding analysis of 100 nM of LayV F (red) or NiV F (orange) to the 4G5 IgG immobilized at the surface of AMC biosensors showing that 4G5 recognizes LayV F but not NiV F. **B-C,** Assessment of cell-cell fusion mediated by LayV F/G (B) or MojV F/G (C) in presence of varying concentrations of the 4G5, 2B2 or D61 (anti HIV gp41, NC:

negative control) IgGs. Effector Neuro-2a cells were co-transfected with LayV F, LayV G and a DSP 1-7 plasmid. Target HEK293T cells were transfected with DSP 8-11. All experiments include 4 biological replicates and error bars correspond to standard deviation (SD).

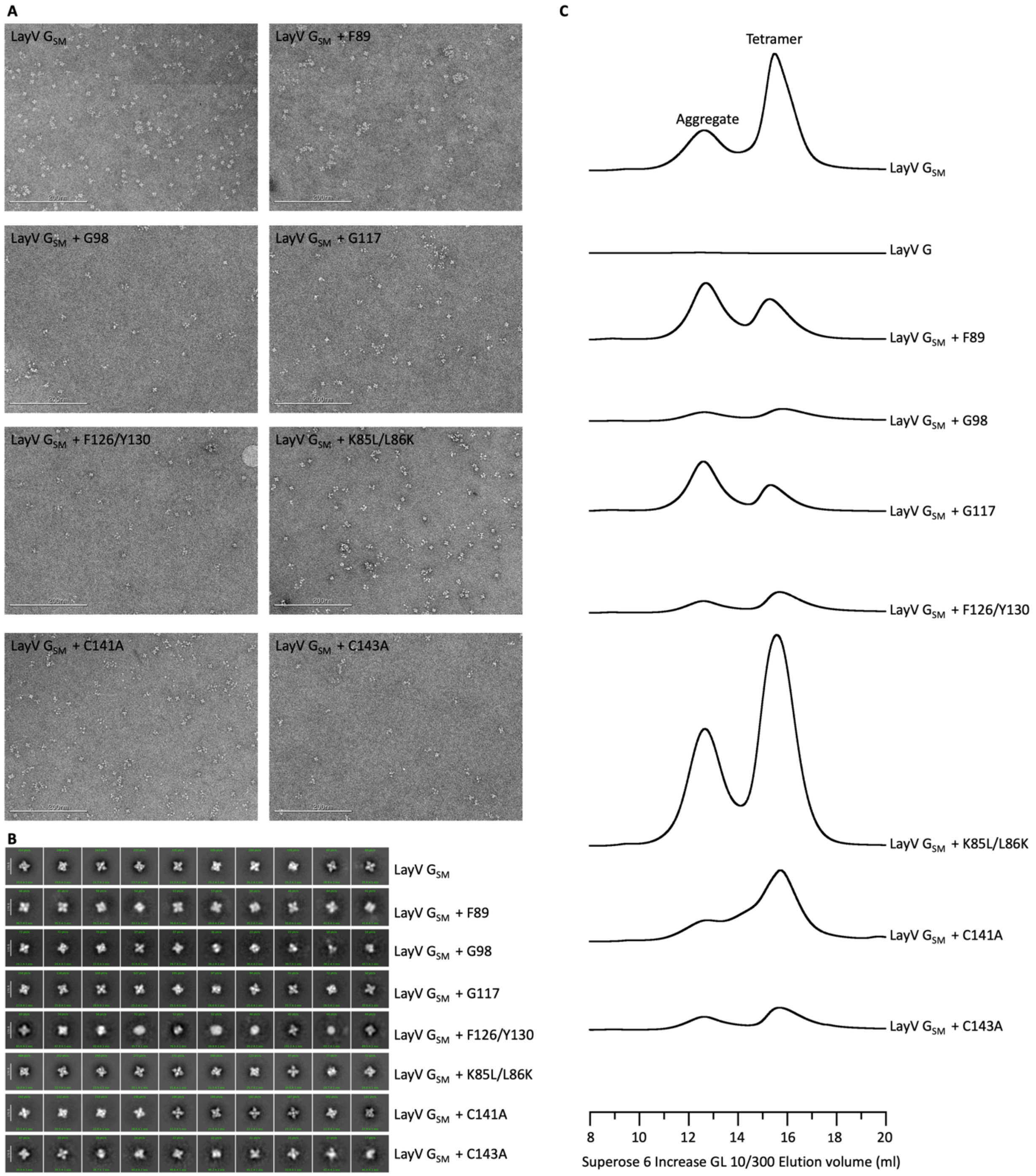

Fig. S9 Characterization of designed LayV G mutants. A, Electron micrographs of negatively stained LayV G mutants after affinity purification and prior to size exclusion chromatography. **B**, 2D class averages obtained from negatively stained LayV G mutants showing the formation of tetramers for all mutants evaluated. Template picking and a prior round of 2D classification was used to enrich well-folded particles. **C**, Size exclusion chromatography profiles of LayV G mutants highlighting variations in protein aggregation and tetramerization.

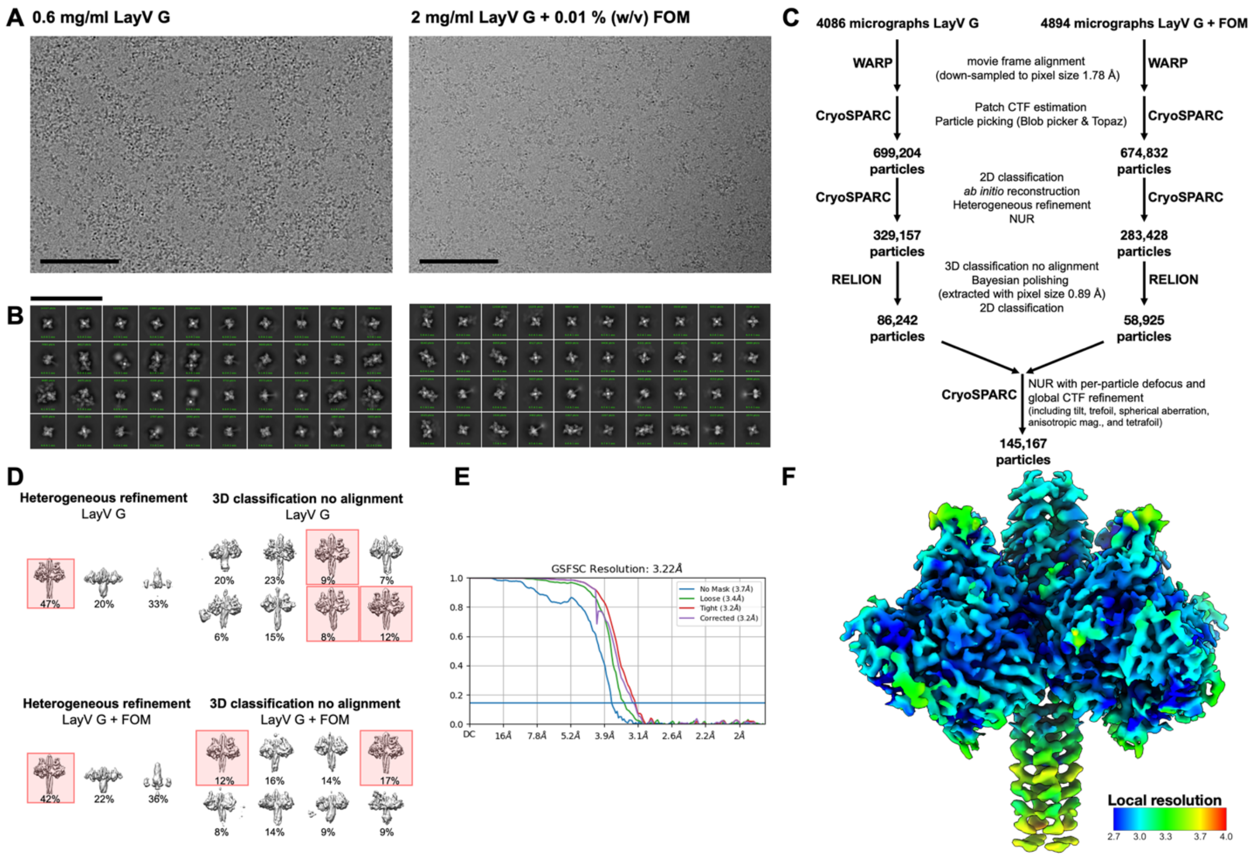

Fig. S10 CryoEM data processing pipeline for LayV G_{SM} harboring the oP4h N-terminal fusion A-B, Representative electron micrograph (A) and 2D class averages (B) of LayV G_{SM} harboring the oP4h N-terminal fusion embedded in vitreous ice. The scale bars represent 100 nm. **C**, Data processing flowchart. CTF: contrast transfer function; NUR: non-uniform refinement. **D**, 3D maps corresponding to 3D classifications with and without alignment referenced in the data processing flow chart. Classes highlighted in red were used for further data processing. **E**, Gold-standard Fourier shell correlation curve for the LayV G reconstruction. The 0.143 cutoff is indicated by the blue line. **F**, Local resolution map calculated using CryoSPARC and plotted onto the sharpened LayV G reconstruction.

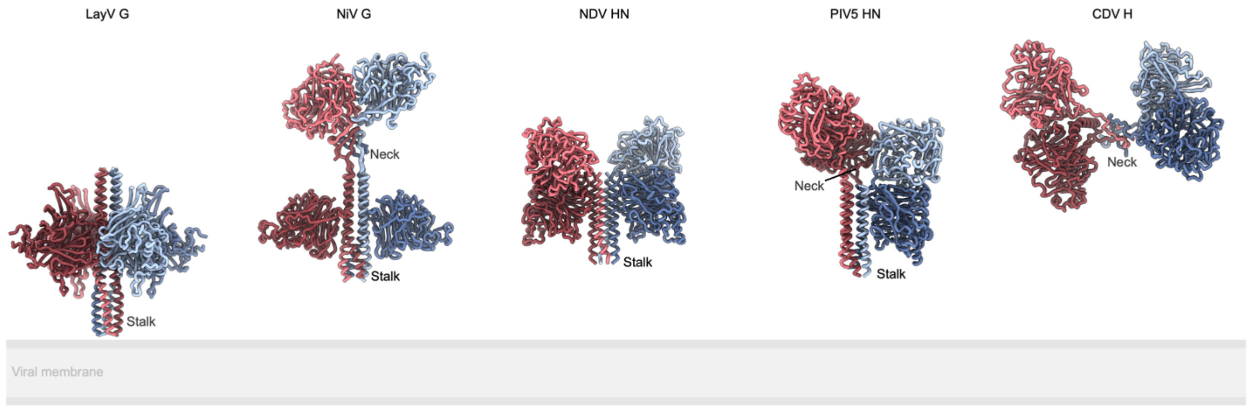

Fig. S11 Comparison of paramyxovirus attachment glycoprotein architectures. Ribbon diagrams of LayV G, NiV G (PDB 7TY0 and 7TXZ), NDV HN (PDB 3T1E), PIV5 HN (PDB 4JF7) and canine distemper virus H (CDV H, PDB 7ZNY) aligned by their stalks to show relative distance from and orientation to the viral membrane.

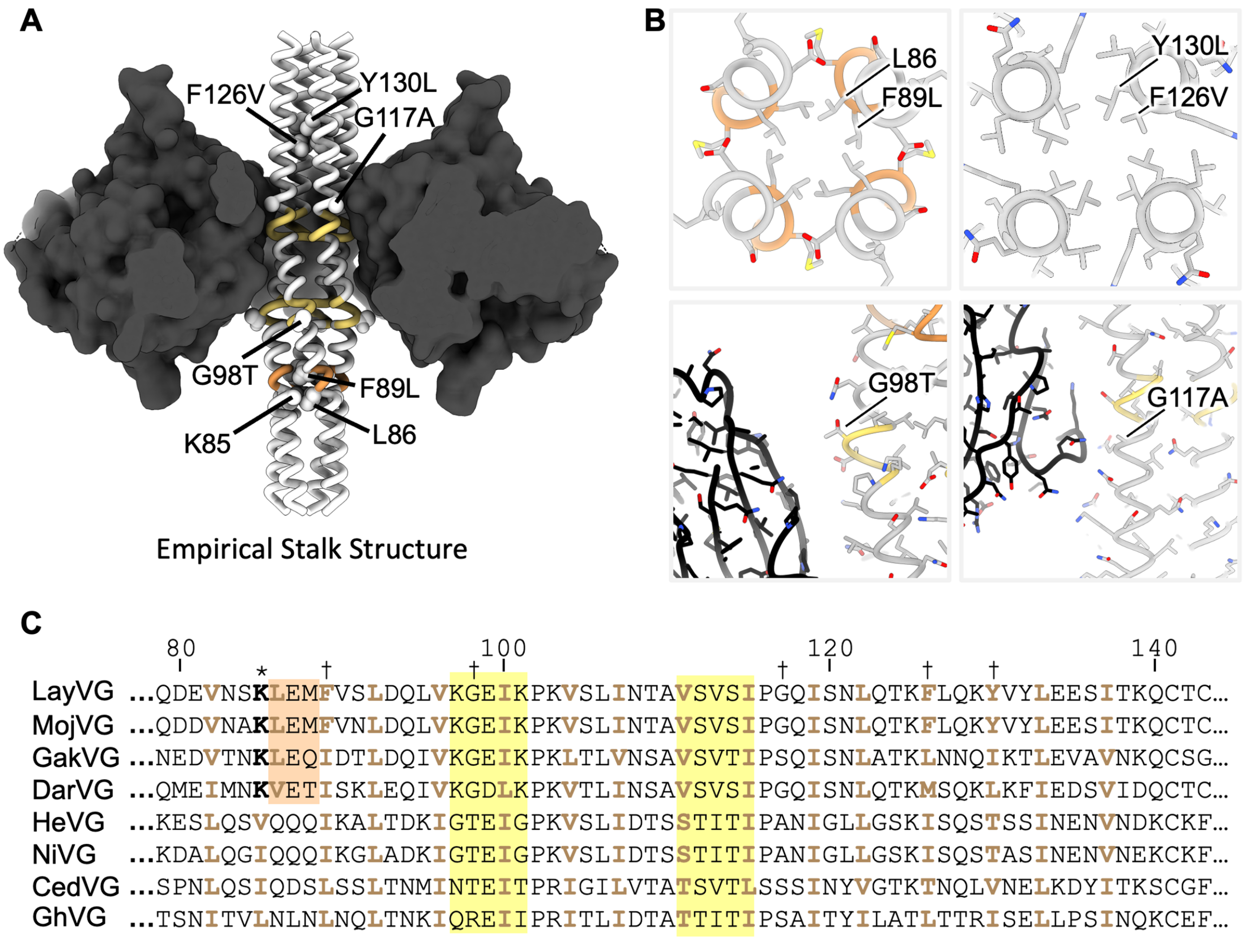

Fig. S12 Stalk stabilizing mutations and comparison of the stalk sequence with other HNV G glycoproteins. A, Designed LayV G stabilizing mutations (labeled white spheres) in the stalk of the LayV G_{SM} cryoEM structure highlighting pi-helices (yellow), and a 3_{10}-helix (orange). The head domains are shown as black surfaces. **B**, Zoomed-in views of stabilizing mutations with relevant and neighboring side chains rendered as sticks colored as in (A). **C,** Amino acid sequence alignment spanning LayV G residues 79-143 with other HNV G glycoproteins. Known or predicted residues facing the interior of the tetrameric coiled coil are shown in beige. Regions with known or predicted pi-helical structures are highlighted in yellow. The region with known or predicted 3_{10}-helical structure is highlighted in orange. K85 is marked with an asterisk. Residues mutated for stability are indicated with a dagger.

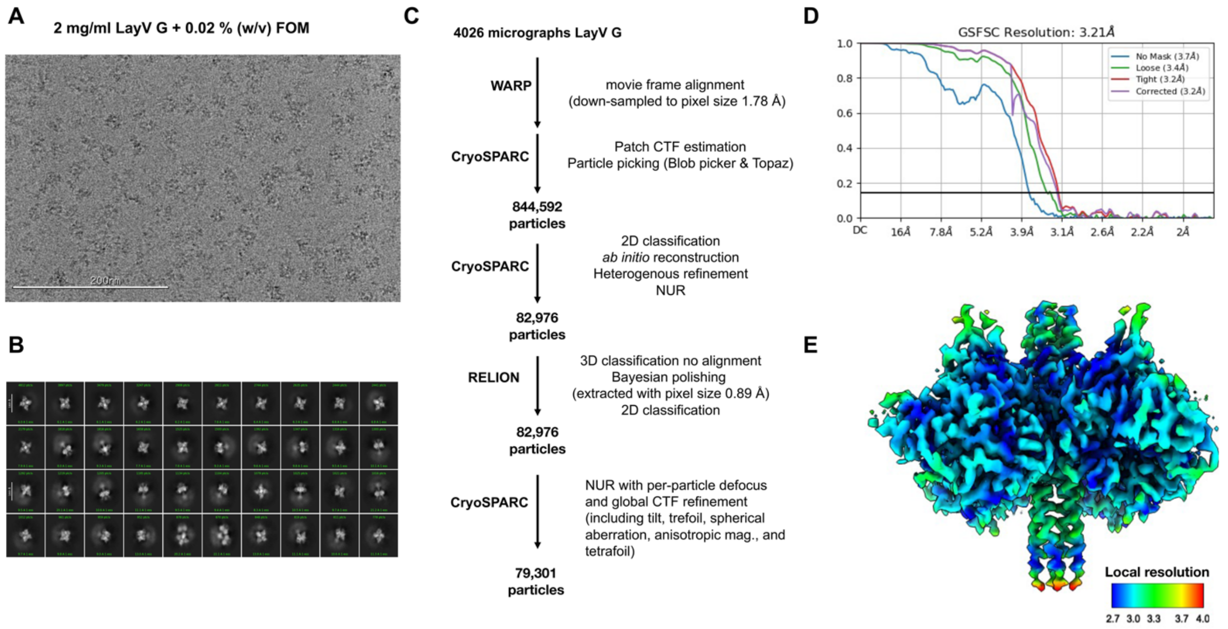

Fig. S13. CryoEM data processing pipeline for LayV G_{SM} harboring the K85L/L86K mutations without oP4h fusion. A-B, Representative electron micrograph (A) and 2D class averages (B) of LayV G_{SM} harboring the K85L/L86K mutations without oP4h fusion embedded in vitreous ice. **C**, Data processing flowchart. CTF: contrast transfer function; NUR: non-uniform refinement. **D**, Gold-standard Fourier shell correlation curve for the LayV G reconstruction. The 0.143 cutoff is indicated by the blue line. **F**, Local resolution map calculated using CryoSPARC and plotted onto the sharpened LayV G reconstruction.

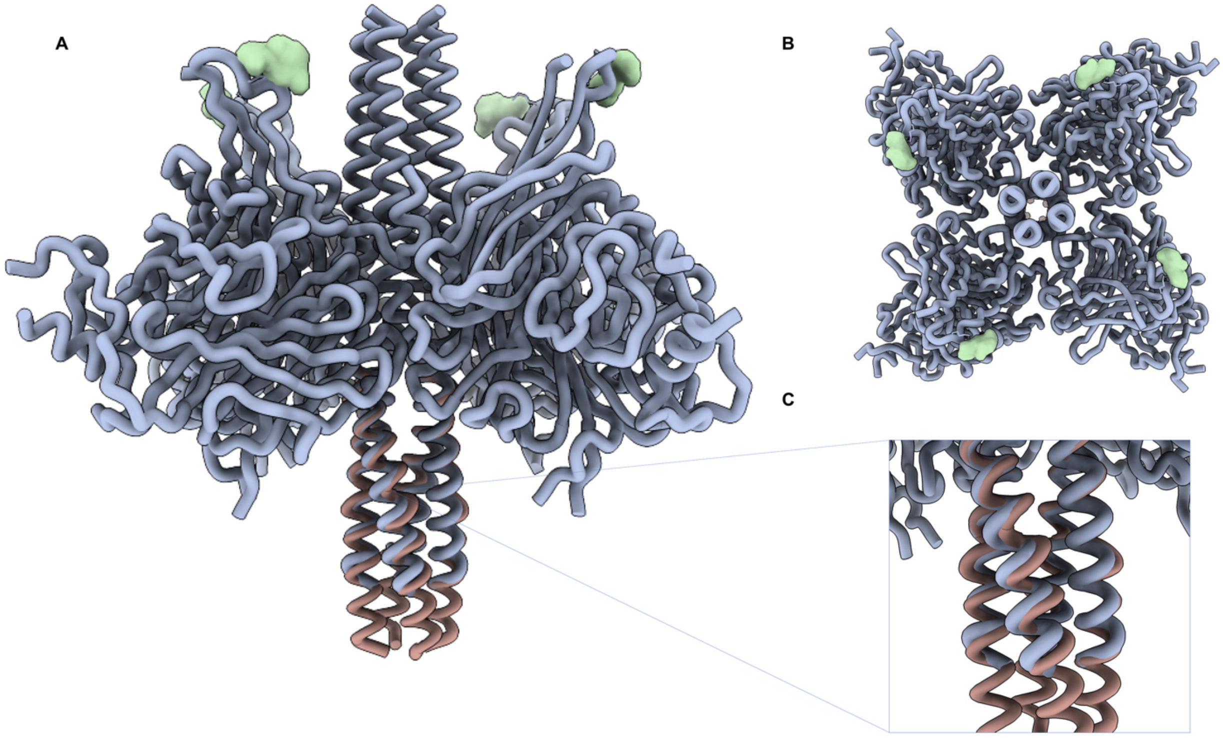

Fig S14. Architectural comparison of LayV G_{SM} harboring the K85L/L86K mutations and LayV G_{SM} with oP4h fusion. A-B. Structural superimposition of LayV G_{SM} with oP4h fusion (red) and LayV G_{SM} harboring the K85L/L86K mutations without oP4h fusion (blue) shown in two orthogonal orientations. **C**. Zoomed-in view showing the absence of 3_{10}-helical structure in LayV G_{SM} with K85L/L86K mutations and retention of the 3_{10}-helix in LayV G_{SM} (with oP4h fusion).

Extended Data video 1. 3DFlex cryoEM density series oscillating between 41 frames showing movement of the LayV G_{SM} (oP4h) stalk.

Table 1 CryoEM data collection and refinement statistics.

	**LayV F prefusion** (EMDB 41640) (PDB 8TVF)	**LayV F postfusion** (EMDB 41639) (PDB 8TVE)	**4G5-bound LayV F (global refinement)** (EMDB 41644)	**4G5-bound LayV F (local refinement proximal)** (EMDB 41642) (PDB 8TVH)	**4G5-bound LayV F (local refinement distal)** (EMDB 41641) (PDB 8TVG)	**GhV F** (EMDB 41636) (PDB 8TVB)	**LayV G_{SM} (oP4h)** (EMDB 41643) (PDB 8TVI)	**LayV G_{SM} K85L/L86K** (EMDB EMD-43593) (PDB 8VWP)
**Data collection and processing**								
Magnification	105,000	45,000	105,000	105,000	105,000	105,000	45,000	45,000
Voltage (kV)	300	200	300	300	300	300	200	200
Electron exposure (e–/Å^2)	63	47	63	63	63	63	47	47
Defocus range (μm)	0.7 - 1.7	0.7 - 1.7	1.3 - 1.7	1.3 - 1.7	1.3 - 1.7	0.5 - 2.5	0.5 - 2.4	0.5- 2.5
Pixel size (Å)	0.843	0.89	0.843	0.843	0.843	0.843	0.89	0.89
Symmetry imposed	C3	C3	C3	C3	C3	C3	C4	C4
Initial particle images (no.)	295,258	681,389	283,620	283,620	283,620	349,279	1,374,036	844,969
Final particle images (no.)	53,917	86,749	60,440	60,440	60,440	60,207	145,167	79,301
Map resolution (Å)<br>FSC threshold	2.5<br>0.143	3.9<br>0.143	3.5<br>0.143	3.4<br>0.143	3.2<br>0.143	2.9<br>0.143	3.2<br>0.143	3.2<br>0.143
**Refinement**								
Model resolution (Å)<br>FSC threshold	2.7<br>0.5	3.9<br>0.5		3.7<br>0.5	3.5<br>0.5	3.1<br>0.5	3.4<br>0.5	3.5<br>0.5
Model resolution range (Å)								
Map sharpening *B* factor (Å^2)	-86	-137	-118	-104	-98	-108	-297	-117
Model composition<br>Non-hydrogen atoms<br>Protein residues<br>Ligands	 10,461<br>1,296<br>12	 9,090<br>1,227<br>6		 6,744<br>939<br>3	 8,310<br>1,131<br>6	 9,687<br>1,266<br>12	 16,120<br>2,056<br>8	 14,820<br>2,012<br>4
B factors (Å^2)<br>Protein<br>Ligand	 8.6<br>17.6	 29.04<br>45.84		 24.49<br>32.77	 22.18<br>38.41	 14.59<br>19.70	 23.19<br>41.31	 22.04<br>43.03
R.m.s. deviations<br>Bond lengths (Å)<br>Bond angles (°)	 0.014<br>1.305	 0.01<br>1.43		 0.01<br>1.29	 0.01<br>1.24	 0.009<br>1.024	 0.01<br>0.94	 0.01<br>1.36
Validation<br>MolProbity score<br>Clashscore<br>Poor rotamers (%)	 0.74<br>0.75<br>0.28	 1.19<br>3.48<br>0.33		 1.00<br>1.41<br>0.52	 1.03<br>1.16<br>0.75	 0.9<br>1.55<br>0.00	 1.01<br>1.63<br>0.00	 0.96<br>1.40<br>0.00
Ramachandran plot<br>Favored (%)<br>Allowed (%)<br>Disallowed (%)	 98.36<br>1.40<br>0.23	 97.77<br>2.23<br>0.00		 97.38<br>2.30<br>0.33	 96.78<br>2.68<br>0.54	 98.56<br>1.20<br>0.24	 97.54<br>2.26<br>0.20	 97.57<br>2.23<br>0.20
